# Rotaxane rings promote oblique packing and extended lifetimes in DNA-templated molecular dye aggregates

**DOI:** 10.1038/s42004-021-00456-8

**Published:** 2021-02-18

**Authors:** Matthew S. Barclay, Simon K. Roy, Jonathan S. Huff, Olga A. Mass, Daniel B. Turner, Christopher K. Wilson, Donald L. Kellis, Ewald A. Terpetschnig, Jeunghoon Lee, Paul H. Davis, Bernard Yurke, William B. Knowlton, Ryan D. Pensack

**Affiliations:** 1 Micron School of Materials Science & Engineering, Boise State University, Boise, ID 83725 USA; 2 SETA BioMedicals, Urbana, IL 61801 USA; 3 Department of Chemistry & Biochemistry, Boise State University, Boise, ID 83725 USA; 4 Department of Electrical & Computer Engineering, Boise State University, Boise, ID 83725 USA

**Keywords:** Optical materials, Organizing materials with DNA, Interlocked molecules

## Abstract

Molecular excitons play a central role in natural and artificial light harvesting, organic electronics, and nanoscale computing. The structure and dynamics of molecular excitons, critical to each application, are sensitively governed by molecular packing. Deoxyribonucleic acid (DNA) templating is a powerful approach that enables controlled aggregation via sub-nanometer positioning of molecular dyes. However, finer sub-Angstrom control of dye packing is needed to tailor excitonic properties for specific applications. Here, we show that adding rotaxane rings to squaraine dyes templated with DNA promotes an elusive oblique packing arrangement with highly desirable optical properties. Specifically, dimers of these squaraine:rotaxanes exhibit an absorption spectrum with near-equal intensity excitonically split absorption bands. Theoretical analysis indicates that the transitions are mostly electronic in nature and only have similar intensities over a narrow range of packing angles. Compared with squaraine dimers, squaraine:rotaxane dimers also exhibit extended excited-state lifetimes and less structural heterogeneity. The approach proposed here may be generally useful for optimizing excitonic materials for a variety of applications ranging from solar energy conversion to quantum information science.

## Abstract (web summary)

DNA templating is a useful strategy to control the positioning and aggregation of molecular dyes on a sub-nanometer scale, but sub-angstrom control is desirable for the precise tailoring of excitonic properties. Here, the authors show that templating squaraine dyes functionalized with rotaxane rings promotes an elusive oblique packing arrangement and extended excited-state lifetimes.

## Introduction

**Fig. 1:**
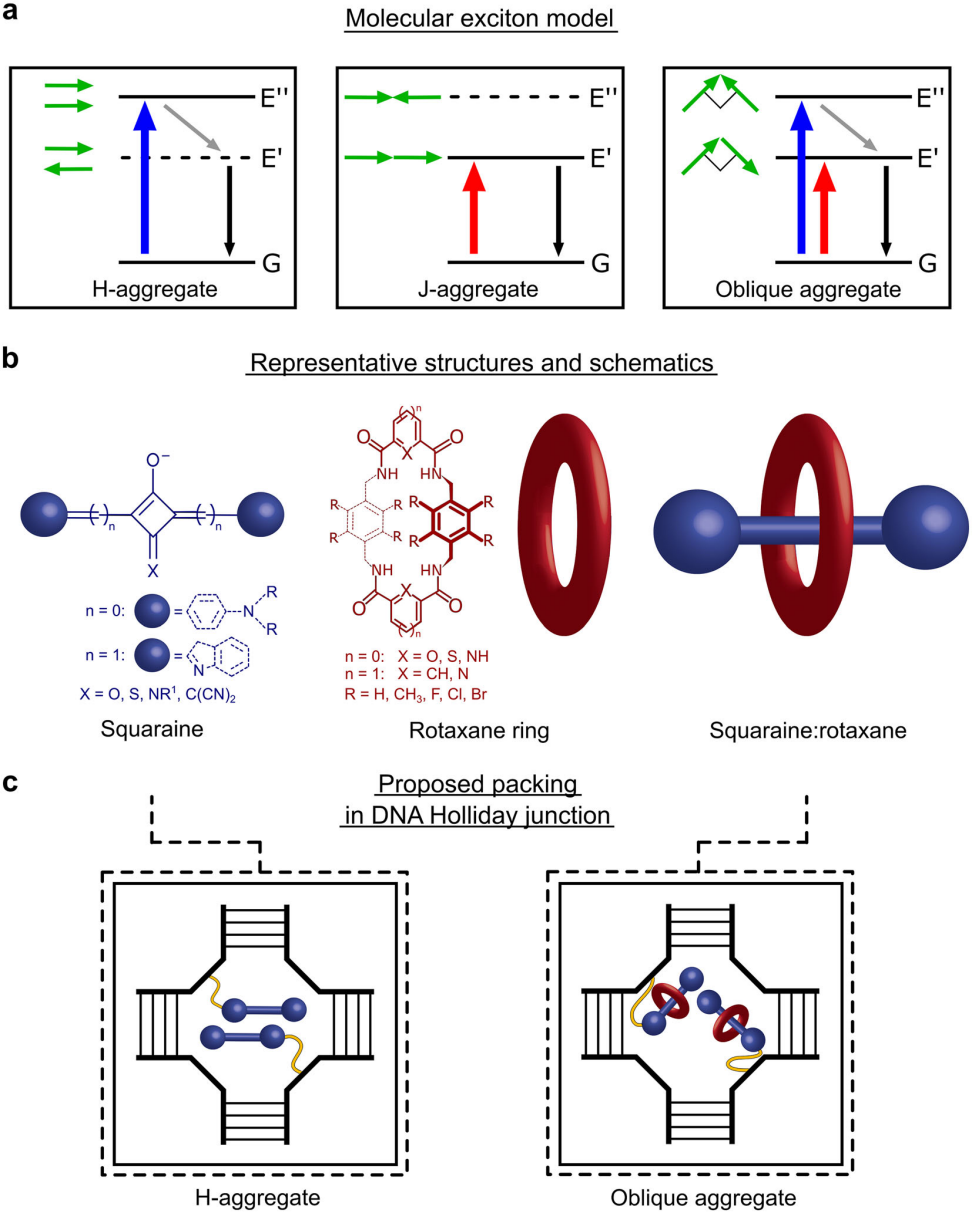
Electronic structure of dimer aggregates, dye structures and schematics, and proposed packing arrangements for SQ and SR dimers templated in a DNA Holliday junction. **a** Energy-level diagrams for an excitonically coupled dimer, where *G*, *E*’, and *E*” denote ground, low-lying, and high-lying excitonic states, respectively. Solid and dashed horizontal lines indicate optically accessible and inaccessible states, respectively. Adjacent to each state is a schematic of the associated dye transition dipole moment orientations. Colored, gray, and black arrows indicate optical absorption transitions, non-radiative transitions, and transitions to the ground state, respectively. **b** Representative structures for an SQ dye, a rotaxane ring, and an SR composite molecule. The exact chemical structures of the SQ dye and SR composite molecule are proprietary. The SQ structure (left) can be aniline-based, where six-membered rings are bound to the central squarate moiety, or it can incorporate substituents such as indolenines, where a five-membered ring is conjugated with the central squarate moiety via a methine bridge. The rotaxane ring (middle) forms hydrogen bonds with the squarate moiety via its amide groups. The rotaxane ring is visualized as a red toroid shape. The SR (right) represents a composite molecule combining the SQ dye and a rotaxane ring. Both SQ and SR dyes are tethered to the DNA backbone via an amide bond between the dye and a non-nucleosidic serinol linker (Supplementary Note 1). **c** Proposed packing for dimers of SQs and SRs templated using a four-armed DNA Holliday junction. Dashed boxes separately highlight the H-aggregate packing proposed for the SQ dimer and the oblique aggregate packing proposed for the SR dimer. Note that the schematic is primarily intended to depict the proposed aggregate packing arrangements, not the conformation of the specific DNA Holliday junction.

Molecular excitons play a central role in a variety of applications, including natural and artificial light harvesting^1–6^, organic optoelectronics^7–9^, and nanoscale computing^10–17^. The utility of excitons in these applications depends on their structure and dynamics^18,19^, which, in turn, are sensitively governed by molecular packing. The molecular exciton model proposed by Kasha^20,21^, which predicts excitonic structure and dynamics via the relative orientation of constituent molecular transition dipole moments (TDMs), describes three limiting cases (Fig. 1a). Most commonly, two molecular TDMs orient in a face-to-face arrangement denoted colloquially as an “H-aggregate.” For an H-aggregate, radiative transitions involving the low-lying excitonic state are forbidden, which results in a blueshifted absorption spectrum (relative to that of the molecules prior to aggregation) and strongly suppressed fluorescence emission. It is also common for two molecular TDMs to orient in an end-to-end, or “J-aggregate,” arrangement. For a J-aggregate, radiative transitions involving the low-lying excitonic state are allowed, which results in a redshifted absorption spectrum and enhanced fluorescence emission. Because applications involving molecular excitons generally depend on energy transfer, and because efficient energy transfer requires appreciable overlap of absorption and emission bands and high emissivity, J-aggregates are generally considered more favorable than H-aggregates. A third type of packing arrangement—the least commonly encountered^22,23^—occurs when the molecular TDMs orient orthogonal to one another in the form of what Kasha^20,21^ labeled as an “oblique aggregate.” A key feature of an oblique aggregate is equal intensity excitonically split absorption bands. For this reason, oblique aggregates are promising candidates for applications that would benefit from panchromatic absorption, such as solar energy conversion^8,9,23–25^ and those that involve excitonic entanglement^26^. In practice, molecular packing also influences exciton dynamics as aggregation in many cases introduces additional non-radiative decay pathways^27,28^ that lead to significant quenching of the excited state, which is undesirable for most excitonic applications. Thus it is important to develop ways of controlling molecular packing to tailor and optimize the photophysical behavior of molecular excitons for specific applications of interest.

In the solution phase, there are several ways of forming molecular (e.g., dye) aggregates. Originally, spontaneous self-aggregation was shown to be a viable means of aggregating dyes in solution^29,30^. However, spontaneous aggregation confers limited control over dye packing. Nature has developed a means of overcoming this challenge by utilizing proteins to template dye aggregation^4^. While significant advances have been made in understanding protein folding^31–35^, predicting high-order protein structures with one or more dye molecules added remains challenging. The use of nucleic acids such as deoxyribonucleic acid (DNA), on the other hand, is an attractive alternative due to the comparatively simpler design rules of nucleotide base pairing. Furthermore, DNA self-assembly gives one the opportunity to bring dyes, which normally would not aggregate, together to form well-defined aggregates. While dye aggregates have traditionally been formed by the adsorption of dyes on duplex DNA or in between base pairs within the duplex^36–40^, recent advances in DNA nanotechnology have made even finer control of dye aggregation possible. Specifically, the covalent attachment, or tethering, of dyes directly to DNA^27,28,41–48^ enables one to control the exact number of dyes being aggregated along with the precise position of dyes at specific nucleotide sites, which allows for spatial tuning in increments as small as ~3.5 Å (i.e., the distance between base pairs). The method could also facilitate aggregation within the cavity of higher-order DNA nanostructures^49^, such as four-armed Holliday junctions (HJs). By tethering dyes directly to DNA and aggregating them using a DNA HJ, additional control of dye packing may be possible.

To date, several approaches have been shown to confer some level of control over packing in DNA-templated and covalently tethered dye aggregates. The approaches generally tune between the most common packing types, H- and J-aggregates, and include altering local base pairs near the dyes, varying the high-level order of the DNA nanostructure, and changing the dye type. For example, Cunningham et al. showed using a duplex DNA template that local base pairs can be modified to tune between H- and J-type packing in dimers of cyanine dyes^27^. Furthermore, Cannon et al. demonstrated that the packing of cyanine dimers templated using duplex DNA, which adopted J-type packing, could be switched to H-type packing by varying the solution salt concentration, which caused the duplexes to form HJs and cyanine tetramers^43^. The same authors showed that by aggregating the cyanine dyes using a HJ and changing the relative position, i.e., in the form of transverse and adjacent dimers, it is possible to switch between H- and J-type packing, respectively^44^. While the aforementioned studies focused on cyanine dyes, squaraines (SQs) are an alternative class of dyes that exhibit similar optical properties and also have a strong propensity to aggregate. SQs include a central squarate moiety (Fig. 1b), which limits isomerization and offers added stability by reducing the likelihood of further oxidation^50^. Critically, Häner and co-workers showed using duplex DNA and dye dimers that changing from cyanine to SQ caused the packing to change from J- to H-type, respectively^41,42^. It is likely that the central squarate moiety provides additional intermolecular interactions that promote H-type packing. A subsequent study has since confirmed the propensity of SQs to form H-aggregates^47^.

In addition to conferring some level of control over dye packing, SQs are also advantageous because they can be readily functionalized with a rotaxane ring. The rotaxane ring hydrogen bonds with and encapsulates the central squarate moiety (Fig. 1b); the composite molecule is called a squaraine:rotaxane (SR). SRs were designed for enhanced stability and to prevent dye aggregation^51–53^. As a result, there are very few studies of SR dye aggregates, most of which focus on how rotaxane rings prevent aggregation^54,55^. It is reasonable to assume that the bulkiness associated with the rotaxane ring sterically hinders spontaneous self-aggregation of SR dyes in solution. By templating the dyes using a DNA HJ, however, the dyes are forced to aggregate, and it may be possible to leverage the rotaxane rings to enable control of molecular packing on a much finer (<3.5 Å) scale (Fig. 1c). Here we test the hypothesis that functionalizing SQ dyes with a rotaxane ring and aggregating them using a DNA HJ will promote the formation of oblique aggregates (useful for broad absorption and potentially beneficial for excitonic entanglement).

## Results and discussion

### Dye aggregation in DNA nanostructures

**Fig. 2:**
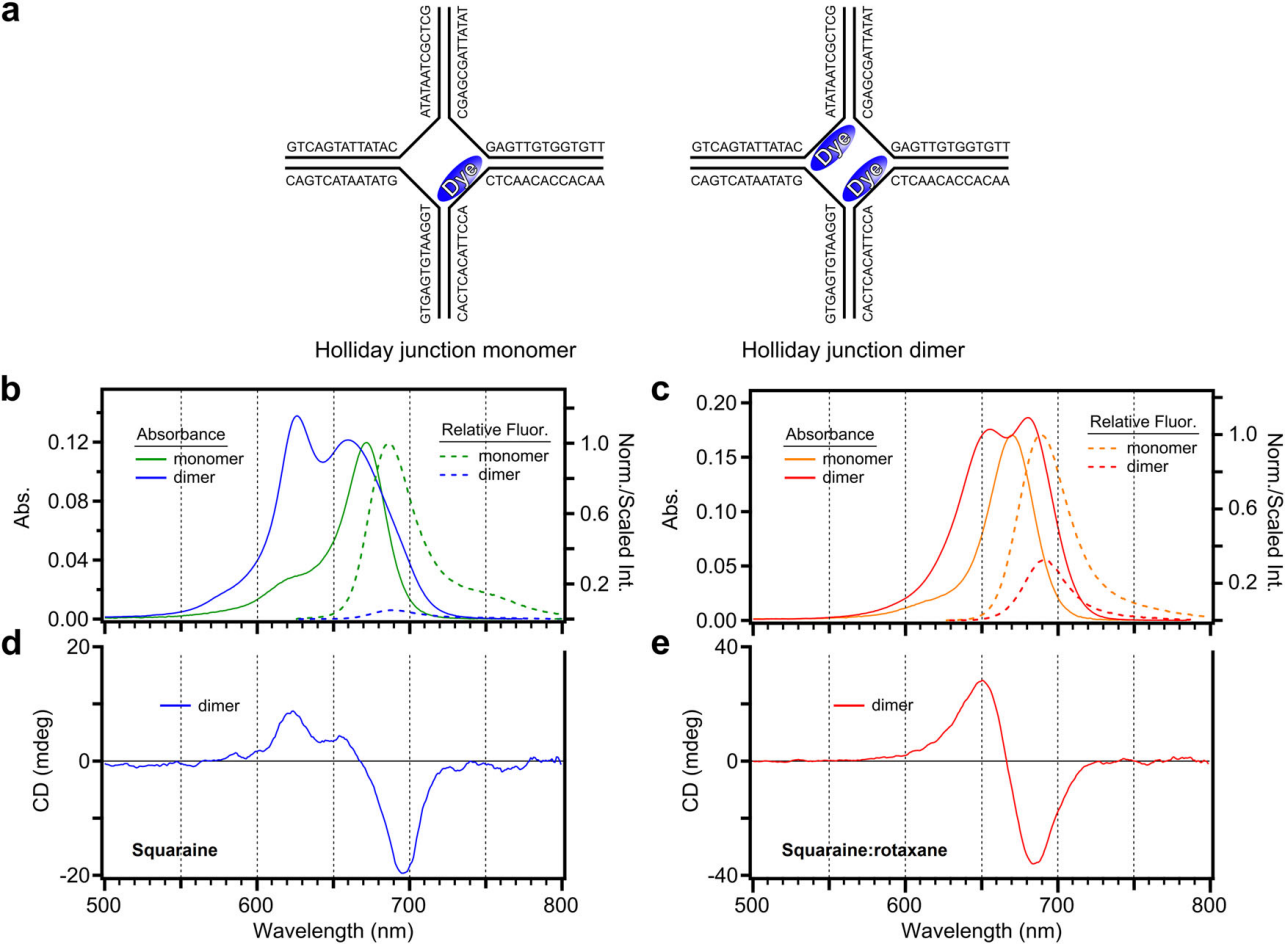
Schematic representation of SQ (left panels) and SR (right panels) dimers templated using a DNA Holliday junction and their corresponding steady-state absorption, fluorescence emission, and CD spectra. **a** Representative schematic of the four-armed DNA Holliday junction used to template the formation of monomers and transverse dimers. The schematic is intended to depict the positions of the attached dyes in the DNA sequences, not the conformation of the Holliday junction. Serinol linkers that attach dyes to the DNA backbone (Supplementary Note 1) are not shown. **b**, **c** Steady-state absorption spectra and fluorescence emission spectra for the SQ and SR dimer solutions. Absorption spectra and fluorescence emission spectra are also shown for the corresponding monomer solutions. To account for differences in the amount of light absorbed by each solution, the measured spectra were divided by the absorptance of each solution at the excitation wavelength. The spectra of the monomer solutions were thereafter normalized to the shortest wavelength band, and the spectra of the corresponding dimer solutions were scaled using the same factor. **d**, **e** CD spectra for the SQ and SR dimer solutions. The CD spectra of the corresponding monomer solutions are achiral and therefore featureless in the same spectral region and have been omitted.

In order to examine how rotaxane rings impact molecular packing in DNA-templated dye aggregates, commercially available SQ and SR dyes were aggregated using DNA HJs. DNA HJs consist of four different oligonucleotide strands designed to self-assemble in solution to form a four-armed structure^44,49^, with each of the arms consisting of duplex DNA. Thermal denaturation experiments were performed and determined that the unlabeled DNA HJs, as well as the DNA HJs labeled with SQ^47^ and SR dyes in the form of a dimer, adopt a stacked conformation where the arms of the four-way junction are oriented coaxially (see Supplementary Note 2). Figure 2a displays a schematic of the DNA HJ configuration used to aggregate the dyes in the form of a monomer and dimer. We specifically chose to examine a “transverse” dimer configuration, where the dyes are attached on opposing strands, based on a previous study by Cannon et al.^44^. These authors showed for a structurally similar cyanine dye that an “adjacent” dimer configuration favored a J-like packing arrangement, whereas an H-like packing arrangement was favored in a transverse dimer configuration. Thus we chose a transverse dimer configuration to investigate whether an H-like packing arrangement is also favored in SQ dimers and whether this packing configuration might be circumvented in SR dimers.

### Monomer optical properties and excited-state dynamics

As the monomer forms the basis of the dimer, the optical properties of SQ and SR monomers were investigated first. Specifically, steady-state absorption and fluorescence emission spectra were measured to characterize the electronic structure of the SQ and SR monomers (Supplementary Note 3). The absorption spectra for the monomer solutions are generally similar (Fig. 2b, c). Each solution exhibits an intense, low-energy absorption band and a second, weaker absorption band at higher energy, which are assigned as 0-0 and 0-1 vibronic transitions, respectively. The 0-0 and 0-1 bands for the SQ monomer are centered at ca. 671 and 620 nm, respectively; for the SR monomer solution, the bands are similarly centered at ca. 670 and 615 nm, respectively. For the SR monomer, the intensity of the 0-1 band relative to that of the 0-0 band is noticeably weaker when compared with the SQ monomer. The fluorescence emission spectra largely mirror the absorption spectra (Fig. 2b, c). Specifically, the fluorescence emission spectrum of the SQ monomer exhibits clear 0-0 and 0-1 emission bands at ca. 686 and 750 nm, respectively. The spectrum of the SR monomer, on the other hand, exhibits only an easily discernible 0-0 band at ca. 688 nm while the position of the lower-energy 0-1 transition is more difficult to assign. This result is consistent with the reduced relative intensity observed in the absorption spectrum. Based on the relative vibronic amplitudes in their respective absorption spectra, we calculate Huang–Rhys vibronic coupling parameters of 0.23 and 0.11 for the SQ and SR monomers, respectively; these values closely match the values of 0.22 and 0.11 that we calculate based on the frequency of the coupled vibrational mode and its displacement^45^, which we obtain from the results of the theoretical modeling in later sections (see Supplementary Note 4). The relatively weak vibronic coupling of the SR monomer, as indicated by the weak intensity observed for the 0-1 vibronic transition and smaller Huang–Rhys parameter of 0.11, is a desirable property because it suggests that vibronic effects are less likely to complicate the excitonic properties of aggregates of these dyes.

### Evidence for oblique packing in SR dimers

We next investigate how the optical properties of the SQ and SR monomers are affected by aggregating the dyes using a DNA HJ. To characterize the electronic structure and molecular packing of the SQ and SR dimers, we performed steady-state absorption and circular dichroism (CD) measurements. Figure 2b shows that the absorption spectrum of the SQ dimer has two main peaks. By visual inspection, the most intense peak is centered at 625 nm with a broader band centered at around 659 nm. Four-component Gaussian fitting of the absorption spectrum identifies the presence of an additional, low-energy band, which peaks at ca. 694 nm (see Supplementary Note 5). The overall hypsochromic shift (i.e., redistribution of oscillator strength to shorter wavelength) relative to the monomer spectrum is a characteristic signature of H-aggregation^20,21^, which is consistent with our expectation of H-aggregation in the SQ dimers. Additionally, we observe that the peak extinction coefficient of the SQ dimer does not differ significantly from that of the SQ monomer (see Supplementary Note 6). Figure 2d displays the CD spectrum of the SQ dimer solution. The SQ dimer CD spectrum exhibits an intense negative feature at around 694 nm, which agrees well with the position of the weak absorption band identified above in the Gaussian fitting analysis. In addition, the SQ dimer CD spectrum exhibits two positive features peaking at ca. 624 and 655 nm. The position of these features is generally consistent with the position of the most intense bands observed in the absorption spectrum (Fig. 2b). The presence of multiple positive features in the CD spectrum of the SQ dimer solution could have several possible origins. The multiple features could arise from dimer structural heterogeneity, i.e., two or more populations of SQ dimer structures exhibiting distinct molecular packing arrangements. Alternatively, the multiple positive features could arise from vibronic effects within a single population of SQ dimer structures. Thus the SQ dimers tend to adopt an H-like packing arrangement, and the SQ dimer solution may consist of either a single population of dimer structures or multiple populations with distinct packing arrangements.

We next examine the photophysics of the SR dimer solution. Figure 2c shows that the absorption spectrum of the SR dimer solution has two prominent bands peaking at around 655 and 681 nm, which are nearly equally blueshifted and redshifted, respectively, compared with the monomer 0-0 absorption band at ca. 670 nm. Remarkably, these absorption bands are also of roughly similar intensity. While the SQ dimers were found to adopt an H-like packing arrangement, the presence of near-equal intensity absorption bands, shifted somewhat symmetrically higher and lower in energy compared with the monomer, suggests that the SR dimers tend to adopt a nearly ideal oblique packing arrangement (Fig. 1a, c). In contrast to the extinction coefficient of the SQ aggregate, the peak extinction coefficient of the SR dimer increases by ca. 39% upon aggregation, which may indicate the presence of overlapping excitonic bands (Supplementary Note 6). To further characterize the photophysics of the SR dimer, CD measurements were performed. Figure 2e displays the CD spectrum of the SR dimer solution. In contrast to the SQ dimer solution, whose CD spectrum is not symmetric and exhibits multiple positive and negative features (Fig. 2d), the line shape in the CD spectrum of the SR dimer solution exhibits near perfect inversion symmetry and lacks additional structure. These observations suggest that, unlike the SQ dimer solution that may be complicated by dimer structural heterogeneity, the packing configuration in the SR dimer solution is largely homogeneous—i.e., the two bands observed in the absorption spectrum arise from a single population of dimer structures. In further support of this interpretation, the SR dimer CD spectrum has two features with peak maxima at ca. 651 and 685 nm, which exhibit excellent agreement with the two bands appearing in the absorption spectrum (Fig. 2c). The agreement confirms that the two absorption bands arise from transitions to the two excitonically coupled states of the dimer. Thus the optical properties of the SR dimers are consistent with an oblique packing arrangement, which supports our hypothesis that templating dyes with rotaxane rings using a DNA HJ may promote such an arrangement.

### Insights on packing and electronic structure from theoretical modeling

**Fig. 3:**
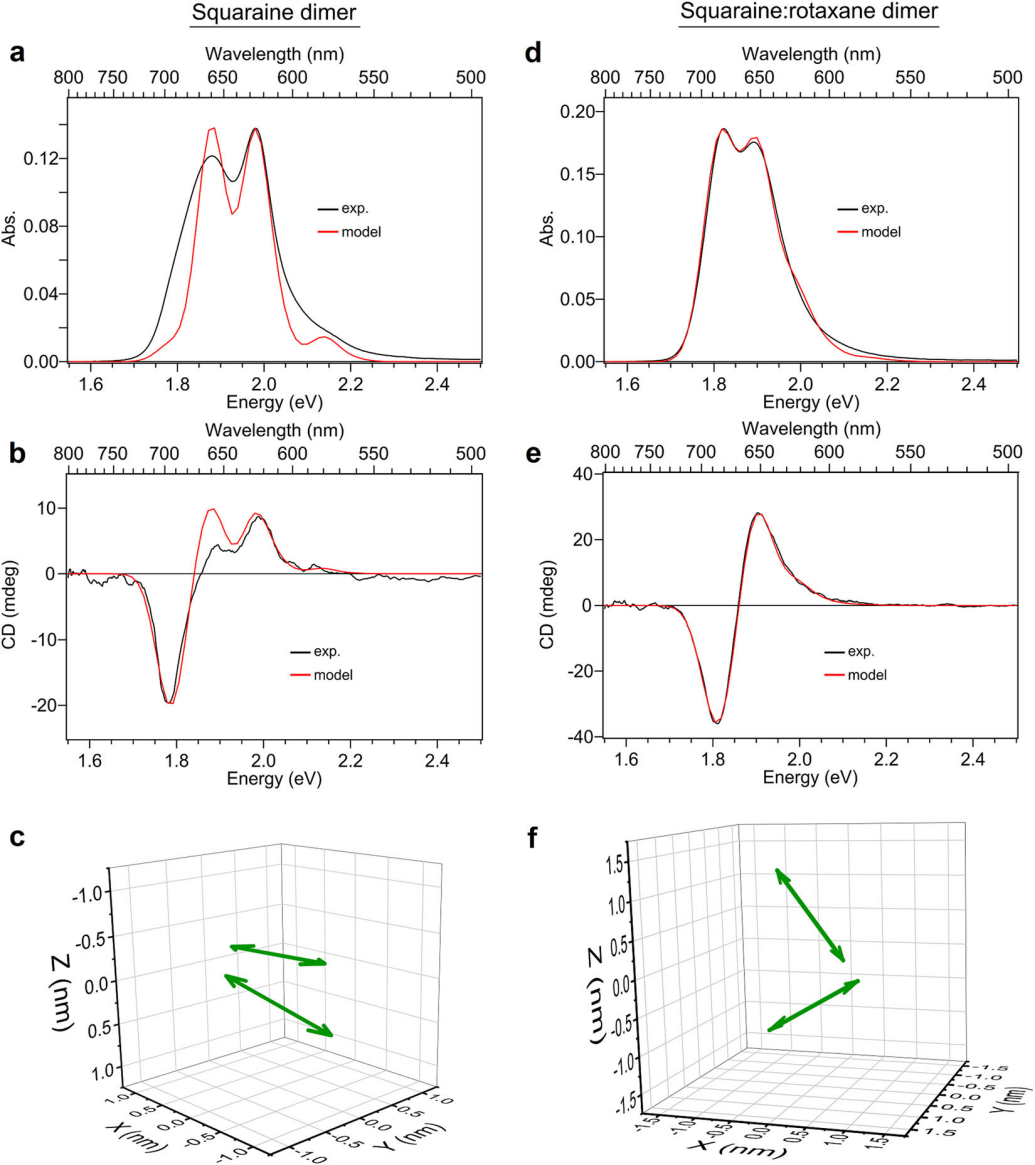
KRM modeling of the dye packing in DNA-templated SQ and SR dimers. **a**, **d** Experimental (black) and simulated (red) absorption spectra for the SQ and SR dimer solutions. **b**, **e** Experimental (black) and simulated (red) circular dichroism spectra for the SQ and SR dimer solutions. **c**, **f** Transition dipole moments (TDMs) derived from the KRM modeling of the absorption and CD spectra. The TDMs corresponding to the two dyes in the dimer structure are shown as double-headed, green arrows, which are assumed to orient along the long axis of the dye. Additional projections of the TDMs along the XY, XZ, and YZ planes are provided in Supplementary Note 4.

To provide further insight into the molecular packing arrangements, the absorption and CD spectra were modeled using an in-house software tool^43,44,47,56^ based on the Kühn–Renger–May (KRM) model^57^. In the analysis, the relative geometries of the TDMs of the dyes are extracted by simultaneously fitting the absorption and CD spectra. Figure 3a, b and Fig. 3d, e show the absorption and CD spectra of the SQ and SR dimer solutions, respectively, along with the simulated spectra for comparison. The simulated spectra exhibit overall good agreement with the experimental results in the case of both SQ and SR dimer solutions. The agreement is much better in the case of the SR dimer solution, which is consistent with the interpretation that the SR dimer solution is more homogeneous compared with the SQ dimer solution. It may be possible to achieve a better fit to the data in a future version of the modeling that accounts for heterogeneity, as was shown in a recent work by Sosa and Wong in modeling the optical absorption spectra of organic thin films^58^. Figure 3c, f plot the relative orientations of the TDMs derived for the SQ and SR dimer solutions, respectively. Figure 3c shows that, as expected, the SQ dimer adopts a face-to-face H-like packing arrangement. In contrast, Fig. 3f shows that, in the SR dimer solution, an H-like packing arrangement is circumvented; rather, the SR dimers adopt an oblique packing arrangement. We attribute the drastically different molecular packing arrangements observed in the case of the SQ and SR dimer structures to the presence of the rotaxane ring. We surmise that the bulky rotaxane rings sterically hinder H-aggregate packing and promote the oblique packing arrangement observed in the SR dimer structures.

**Fig. 4:**
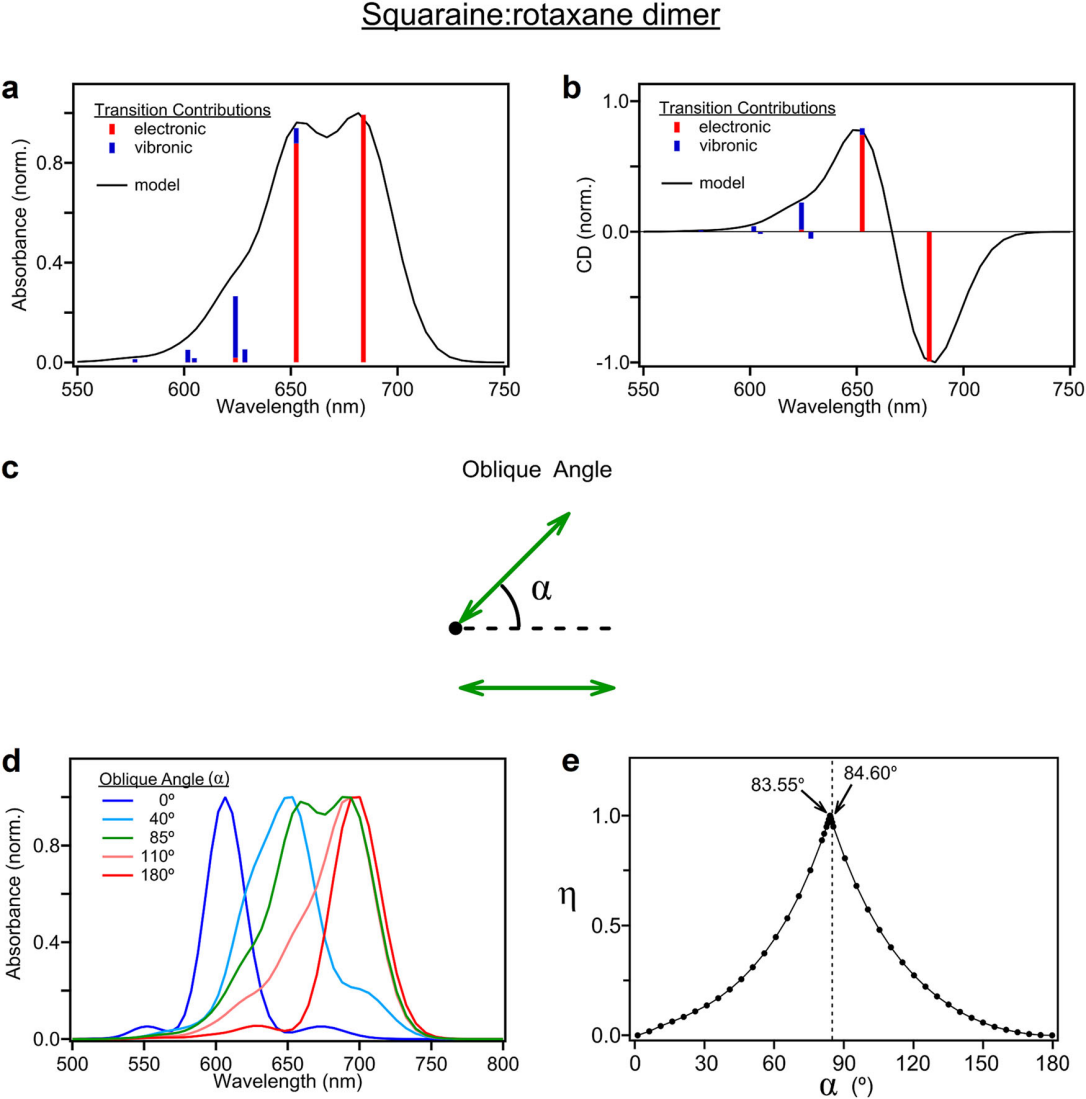
Relative electronic and vibronic contributions to optical spectra and sensitivity of the absorption spectrum to oblique angle. **a**, **b** Electronic and vibronic contributions to the steady-state absorption and CD spectra of the SR dimer structure. **c** Schematic showing the oblique angle between two molecules in a dimer that was varied to produce the absorption spectra in **d**. The TDMs of the molecules are represented with arrows and are assumed to be oriented along the long axis of the molecule. The oblique angle is varied by rotating the TDM around the point indicated by the black circle. The dashed line corresponds to a relative orientation between molecules where *α* = 0°. **d** Simulated absorption spectra as a function of oblique angle, *α*. Dipoles are assumed to be coplanar with a fixed distance of nearest approach, modeled using parameters determined from the experimental data of the SR monomer and dimer solutions. **e** Plot of the ideality factor, *η*, as a function of *α*. When *η* = 1, the electronic bands are of equal intensity; when *η* = 1, the electronic bands exhibit their maximum intensity difference. The dashed black line represents the *α* output from the KRM modeling for the SR dimer.

To gain additional insight into the unique optical properties of the SR dimers, Fig. 4a, b display the simulated absorption and CD spectra along with bar lines representing the excitonic transitions used to model the data. The KRM modeling indicates that the two lowest-energy transitions of the SR dimer are mostly (ca. 99 and 93%, respectively) of electronic origin, i.e., associated with the 0-0 vibronic transition, which is consistent with our expectations of excitonic coupling; in the case of the SQ dimer, heterogeneity complicates a similar analysis (Supplementary Note 7). Remarkably, Fig. 4a shows that the electronic transitions in the absorption spectrum are proximally spaced and of near-equal intensity. This unique feature of the absorption spectrum is advantageous for applications such as excitonic entanglement that require the simultaneous excitation of states^59^. Namely, near-equal intensity excitonic absorption bands will facilitate generating the largest possible electronic coherence signal (Supplementary Note 8), which is optimal for excitonic entanglement. At higher energies (and shorter wavelengths), the KRM modeling derives multiple bar lines of vibronic origin, i.e., associated with 0-n vibronic transitions; these transitions are of low intensity, likely due to the weak vibronic coupling exhibited by the constituent dye molecules (Fig. 2c). As a result, most of the oscillator strength of the electronic band is associated with the excitonic transitions, which is particularly advantageous because the weak vibronic contributions should lead to minimal decoherence of a persistent entangled excitonic state^60,61^.

Given the relative scarcity of ideal oblique packing and near-equal intensity excitonically split absorption bands in molecular dye aggregates^22^, it is useful to consider the range of packing orientations capable of yielding these desirable optical properties. In Fig. 4c, we consider a model system composed of two TDMs, where *α* represents the oblique angle between the vectors, the black circle represents the axis of rotation, and the dashed black line represents the case when *α* = 0° (see Supplementary Note 9 for additional details). Figure 4d illustrates that the unique absorption spectrum of the ideal oblique aggregate (calculated by the model) is reproduced only over a small range of oblique angles. As expected, the absorption spectra generated for angles of 0° and 180° are consistent with the electronic structure of ideal H- and J-aggregates (Fig. 1a), namely, the spectra exhibit a predominant high- and low-energy absorption band, respectively, with the corresponding low- and high-energy absorption bands strongly suppressed. The angles of 40° and 110°, on the other hand, produce absorption spectra consistent with “oblique H” and “oblique J” aggregates, generally the two most prevalent dye aggregate packing arrangements^27,28,41–48^. Much like for ideal H- and J-aggregates, the spectra for oblique H- and J-aggregates exhibit predominantly high- and low-energy absorption bands, respectively; however, the intensity of the corresponding low- and high-energy absorption bands are not strongly suppressed. Lastly, Fig. 4d shows that only a specific angle of ca. 85° is able to reproduce the optical properties of the “ideal” oblique aggregate, namely, near-equal-intensity excitonically split absorption bands.

To better evaluate the range of angles capable of reproducing the oblique aggregate absorption spectrum, we introduce an “ideality” factor, *η*, calculated as the minimum of the two electronic peak heights divided by the maximum of the two electronic peak heights. Defined in this manner, when *η* = 1 the electronic bands are of equal intensity; conversely, the electronic bands have their maximum intensity difference when *η* = 0. The *η* value is 0.970 for the experimental SR dimer results, for which the KRM model calculated an oblique angle of 84.60°. Figure 4e demonstrates that only a narrow range of oblique angles reproduces the ideality of the oblique aggregate absorption spectrum, as is evident by the cusp-like shape of the curve, and that the *η* value of the SR dimer solution is very similar to the ideal *α* angle of 83.55°. We attribute the near-ideal oblique angle of the SR dimer to a combination of tethering the dyes to DNA and the steric repulsion introduced by the rotaxane rings. Taken together with the results above, we conclude that including a rotaxane ring in these dye aggregates promotes a packing arrangement, supported over only a narrow range of oblique angles, that yields optical properties that are potentially optimal for applications such as excitonic entanglement. Further modification of the rotaxane ring, which can encapsulate a range of dyes beyond SQs^51,62,63^, may provide a general means of controlling dye packing on a sub-Angstrom scale.

### Fluorescence emission quenching and excited-state lifetimes

In addition to modifying electronic structure, dye aggregation may also impact exciton dynamics. To aid in understanding the dynamics of the dimers, we first assessed the excited-state dynamics of the monomer solutions. Specifically, we performed fluorescence quantum yield (FQY) and time-correlated single photon counting measurements. We found that the SQ and SR monomer solutions have relatively high FQY values of 0.36 and 0.48, respectively, and each exhibits a fluorescence lifetime of ca. 2.9 ns (see Supplementary Notes 10 and 11). These results indicate that the SQ and SR monomer solutions are highly emissive and exhibit long excited-state lifetimes, which suggests that non-radiative decay plays a minor role in the intrinsic photophysics. Such properties are beneficial in applications that rely on highly efficient energy transfer, such as energy conversion and nanoscale computing.

**Fig. 5:**
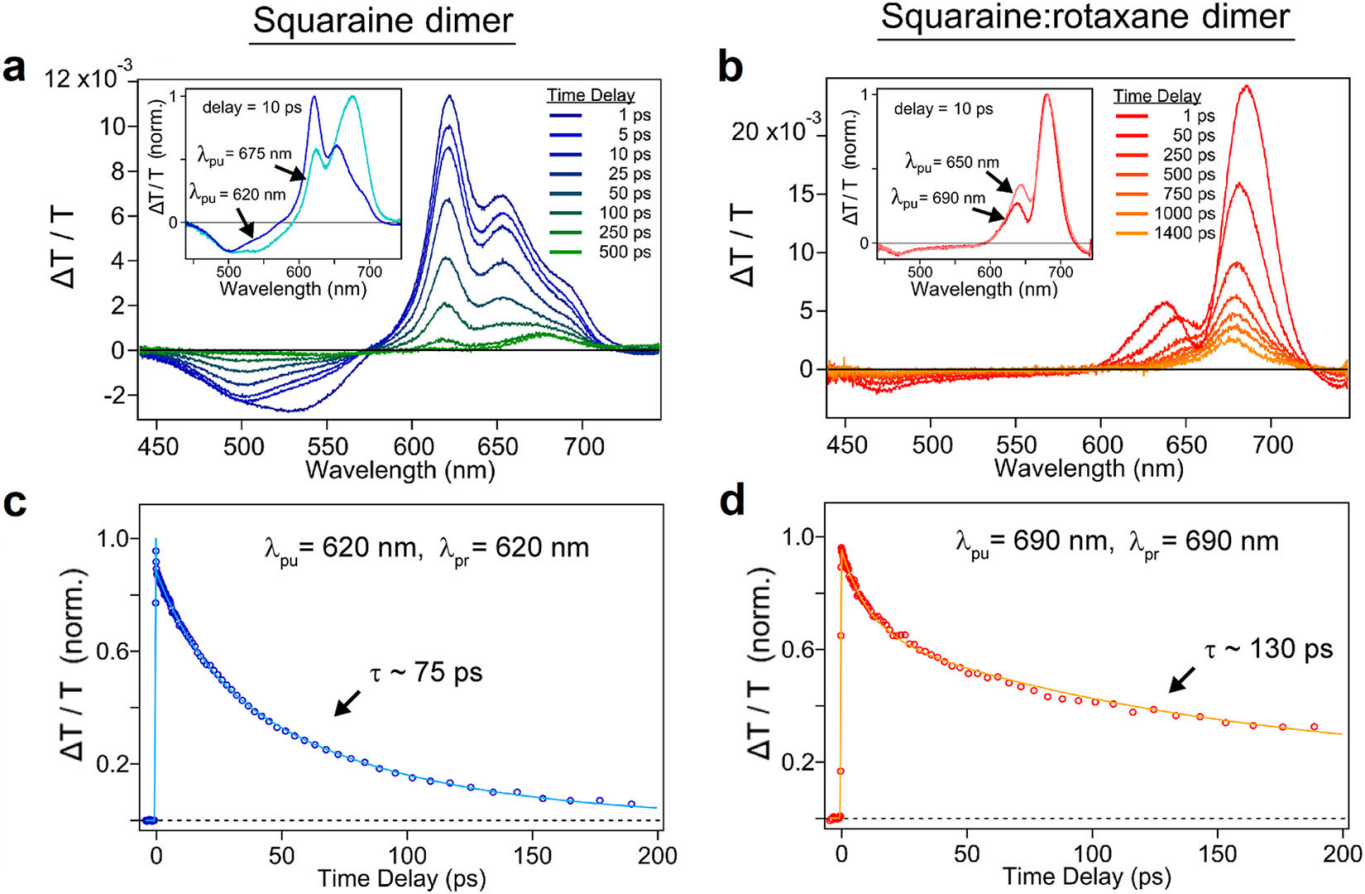
Transient absorption of SQ and SR dyes templated in the form of transverse dimers using a DNA Holliday junction. **a**, **b** Selected TA spectra of the SQ and SR dimer solutions. Experiments were performed with incident pump wavelengths of 620 and 690 nm, respectively. The inset in each panel displays 10 ps spectra obtained for the solutions at two distinct pump wavelengths with comparable pump fluence. **c**, **d** Selected kinetics trace for the SQ and SR dimer solutions obtained with pump wavelengths of 620 and 690 nm, respectively. The probe wavelengths were selected to coincide with the largest amplitude GSB feature appearing in the TA spectra of each solution at ca. 620 and 690 nm, respectively. The kinetics traces have been normalized to their maximum amplitude. A fit from a global target analysis (see Supplementary Note 15) overlays the data in each panel and the lifetime of the corresponding dimer structure is indicated in the plot.

To examine the excited-state dynamics of the dimers, we further performed relative fluorescence emission and femtosecond transient absorption (TA) measurements on the SQ and SR dimer solutions. Figure 2b, c show that significant fluorescence quenching is observed in both SQ and SR dimer solutions; further details can be found in Supplementary Notes 12 and 13. Similar quenching has recently been observed in aggregates of DNA-templated cyanine dyes^27,28,43,44^, which was found to arise from new non-radiative decay pathways introduced upon aggregation^27,28^. Femtosecond TA spectroscopy was subsequently used to directly time resolve the excited-state dynamics. The positive TA signals (represented as Δ*T*/*T*) between 600 and 700 nm are assigned as ground-state bleach (GSB) features for each of the TA spectra in Fig. 5a, b. The differences in the pump-wavelength-dependent GSB signals of the SQ dimer (inset of Fig. 5a) are consistent with our interpretation that the solution is largely a heterogeneous mixture of dimer structures, while the similarities between the GSB signals of the SR dimer (inset of Fig. 5b) demonstrate that the solution is more homogeneous with respect to dimer structures (see Supplementary Notes 14–16 for additional detail). A global target analysis^64,65^ was performed on each spectrotemporal TA dataset (Supplementary Note 15), and the excitonic lifetimes of the SQ and SR dimers were determined to be ≤75 and ca. 130 ps, respectively (Fig. 5c, d). Thus, along with promoting an oblique packing arrangement, including rotaxane rings in dye aggregates may additionally benefit excitonic materials with extended excited-state lifetimes and reduced aggregate heterogeneity.

## Conclusions

In conclusion, we have shown that encapsulating dyes with a rotaxane ring and aggregating them using DNA promotes an uncommon oblique packing arrangement with optical properties optimal for a variety of applications. Specifically, while SQ dimers without rotaxane rings were observed to adopt an H-aggregate packing arrangement, SR dimers exhibited clear optical signatures—including near-equal-intensity excitonically split absorption bands—of an oblique packing arrangement. Additional details of the packing arrangements, including the relative orientation of the dyes, were determined by theoretical modeling of the optical spectra. The theoretical modeling also determined that the two lowest-energy excitonic transitions of the SR dimer are mostly electronic in nature and are of near-equal intensity, which has the potential to be optimal for excitonic entanglement. Using a simple model, we further determined that the ideal oblique aggregate absorption spectrum could only be reproduced over a narrow range of oblique angles. These results provide insight into why such a packing arrangement is so uncommon in molecular aggregates and highlight the inclusion of rotaxane rings in dye aggregates as a potential means to promote this unique packing arrangement. SR dimers also exhibited longer excited-state lifetimes and considerably less structural heterogeneity, which are additional beneficial properties. Thus adding rotaxane rings to dye aggregates may represent a promising approach to optimizing the properties of excitonic materials for applications ranging from solar energy conversion to quantum information science.

## Methods

### Construct synthesis and sample purification

Dye-labeled and unlabeled DNA oligonucleotide strands were purified via dual high-performance liquid chromatography and standard desalting, respectively (Bio-Synthesis, Lewisville, TX). The DNA oligonucleotide strands were labeled internally with a SQ or SR dye through covalent attachment via a non-nucleosidic serinol-based linker (Supplementary Note 1). The dyes selected for this study were an indolenine-based SQ dye, Square-660-NHS (K8-1352; SETA BioMedicals, Urbana, IL), and an aniline-based SR dye, SeTau-670-NHS (K9-4169; SETA BioMedicals, Urbana, IL). All DNA oligomers were rehydrated in ultrapure water generated using a Barnstead Nanopure system (Thermo Scientific, Waltham, MA) to prepare 53 μM stock solutions. Concentrations of DNA samples were determined using a NanoDrop One Microvolume UV-Vis Spectrophotometer (Thermo Scientific, Waltham, MA) using the extinction coefficients at 260 nm provided by the manufacturer. DNA HJs were prepared by combining equimolar amounts of complementary strands in 1× TBE and 15 mM MgCl_2_ buffer solution^44^ to a final DNA concentration of about 1.5 μM for monomer solutions and 2.0 μM for the dimer solutions. Supplementary Note 17 provides evidence indicating that the SR composite molecule is stable after attachment to the oligonucleotide and after formation of the DNA HJ.

### Steady-state absorption spectroscopy

Absorption spectra were measured using a Cary 5000 UV–Vis–NIR spectrophotometer (Agilent Technologies, Santa Clara, CA). Samples were contained in a 1-cm path length quartz cuvette (Starna Cells, Atascadero, CA). Absorption spectra were measured using a step size of 1 nm.

### Steady-state fluorescence spectroscopy

Steady-state fluorescence emission spectra were measured using a Horiba Fluorolog 3 spectrofluorometer (Horiba Scientific, Edison, NJ). Five measurements were obtained and averaged to improve the signal-to-noise ratio. Samples were contained in a 1-cm path length quartz cuvette (Starna Cells, Inc., Atascadero, CA). Sample solutions were diluted to ensure that the optical density of the solution was <0.05 within the spectral range from 500 to 800 nm. Fluorescence emission spectra were measured using a 1-nm step size, 0.5-s integration time, and an excitation wavelength of 615 nm. The spectra were corrected for the wavelength dependence of the detection system response using the correction curve provided by the manufacturer. To account for differences in the amount of light absorbed by each solution, the corrected spectra were divided by the absorptance (i.e., the fraction of light absorbed) of each solution at the excitation wavelength.

FQYs were determined by measuring the fluorescence emission spectrum of a sample solution and comparing that with the fluorescence emission spectrum of a standard reference material with a known quantum yield (Φ_ref_)^66^. Measurement parameters, including excitation wavelength and slit widths (chosen to maximize signal for the relative standard while remaining within the linear range of the detection system) were held constant between measurements. The sample FQY (Φ_smpl_) was evaluated according to Eq. (1),

1 $$\Phi _{{\mathrm{smpl}}{\mathrm{.}}} = \Phi _{{\mathrm{ref}}{\mathrm{.}}}\left( {\frac{{I_{{\mathrm{smpl}}{\mathrm{.}}}}}{{I_{{\mathrm{ref}}{\mathrm{.}}}}}} \right)\left( {\frac{{A_{{\mathrm{ref}}{\mathrm{.}}}^\lambda }}{{A_{{\mathrm{smpl}}{\mathrm{.}}}^\lambda }}} \right)\left( {\frac{{n_{{\mathrm{smpl}}{\mathrm{.}}}^2}}{{n_{{\mathrm{ref}}{\mathrm{.}}}^2}}} \right)$$

where $I_{{\mathrm{smpl}}{\mathrm{.}}}$ and $I_{{\mathrm{ref}}{\mathrm{.}}}$ are the integrated fluorescence emission intensities of the sample and reference solutions, respectively, the $A^\lambda$ terms are the absorbances of the corresponding solutions (in units of optical density) at the excitation wavelength, and the $n$ terms are the refractive indices of the solutions. The FQYs of the Square 660 and SeTau 670 monomer solutions were determined using a solution of Cy5 monomer as a relative standard. Specifically, the FQY of Cy5 monomer templated using single-stranded DNA (and dissolved in 1× TAE buffer with 0 mM MgCl_2_) was previously measured to be 0.29^28^. It was assumed that TAE and TBE buffer solvents have nearly identical refractive indices; therefore, the right-most term of Eq. 1 was set equal to 1. FQY values for the Square 660 and SeTau 670 dimer solutions were obtained separately, using an excitation wavelength of 650 nm and using the FQY values for monomer solutions as a relative standard.

### CD spectroscopy

CD spectra were obtained from a J-810 spectropolarimeter (JASCO, Easton, MD). Samples were measured in a 1-cm path length micro quartz cuvette (JASCO 0553, 100 μL capacity). The CD signal was monitored at room temperature with excitation ranging from 200 to 800 nm. The signal was averaged over three scans with a scan rate of 200 nm min^−1^.

### Theoretical modeling

Following previous work^43,44,47,56^, dimers were modeled using an in-house developed software (version 12.5) utilizing the KRM method. The software treats the dominant vibronic mode and diagonalizes the Holstein Hamiltonian^57^ to simultaneously fit absorbance and CD data in order to extract relative orientations and coupling information. Dyes are modeled as extended transition dipoles aligned with the long axis of each dye. The model assumes that the dimer solution is homogeneous and that charge transfer coupling can be neglected. The fitting algorithm proceeds via stochastic search in which an aggregate configuration is successively randomly mutated to see if the resulting computed absorbance and CD spectra provide a better fit to the data as determined by the mean-square deviation.

### Femtosecond TA spectroscopy

Femtosecond TA measurements were performed with a 1-kHz regeneratively amplified Ti:sapphire laser system (Coherent Legend Elite, Santa Clara, CA) that delivers ~45 fs pulses at ~800 nm with an average power of ~3 W. Pump and probe beam paths were generated by placing a beamsplitter at the output of the laser amplifier. A large fraction of the power was used to drive an optical parametric amplifier (Coherent OPerA Solo, Santa Clara, CA) to convert the 800 nm light to *λ*_pump_, the pump wavelengths used for the femtosecond TA measurements. The pump beam was measured to have a pulse duration of ca. 180 fs, as shown in Supplementary Note 18. The probe beam was generated by focusing a small fraction of the 800 nm light into a sapphire optical flat (Newlight Photonics, Ontario, Canada). The resultant white-light continuum (WLC) subsequently passed through a 750-nm short-pass filter (Thorlabs, Newton, NJ) to isolate the WLC from the 800 nm fundamental from the amplifier. A spectrum of the probe beam, which spanned from ca. 440 to 750 nm, is shown in Supplementary Note 19. The pump and probe beams were routed to and spatially overlapped at the sample position. To control the temporal overlap, the path length of the pump beam was varied using an ALS 20000 series Aerotech delay stage (Aerotech, Pittsburgh, PA) capable of a path length difference up to 300 mm and with a minimum step size of 1 μm. The intensity of the probe beam as a function of wavelength after the sample was measured with a spectrograph, which consisted of a Kymera 193i monochromator (Andor, Belfast, Northern Ireland) equipped with a Zyla 5.5 sCMOS detector (Andor, Belfast, Northern Ireland). The relative pump and probe polarization was controlled with a combination of a *λ*/2 waveplate and polarizer in the pump beam path. Measurements were performed with pump and probe polarizations oriented at the magic angle at the sample. The solutions were contained in a 2-mm path length glass spectrophotometer cell (Starna Cells, Atascadero, CA). A magnetic stir bar was included in the spectrophotometer cell and was stirred over the course of the measurement using a magnetic stirrer (Ultrafast Systems, Sarasota, FL). The optical density of the samples varied from ca. 0.1 to 0.2 at the excitation wavelength. Samples were passed through a 0.45-μm polyethersulfone syringe filter (VWR, Radnor, PA) to remove particulates prior to optical measurements. The pump beam spot size was estimated to be ca. 250 μm by measuring the pulse energy before and after being transmitted through a pinhole of known diameter located at the sample position. Pulse energies were measured with an optical power sensor and meter (Coherent, Santa Clara, CA). For measurements on the SQ dimer solutions, pump fluences were 33 and 30 μJ cm^−2^ for incident pump wavelengths of 620 and 675 nm, respectively; for measurements on the SR dimer solutions, pump fluences were 19 and 16 μJ cm^−2^ for incident pump wavelengths of 690 and 650 nm, respectively.

### Supplementary information

Supplementary Information

## Data Availability

The data that support the findings of this study are available from the corresponding author upon reasonable request.
