# Supplementary material for: Rotaxane rings promote oblique packing and extended lifetimes in DNA-templated molecular dye aggregates
Source: Commun Chem. 2021 Feb 18;4:19. doi: 10.1038/s42004-021-00456-8 (PMC9037907; doi:10.1038/s42004-021-00456-8)
Supplement: Supplementary file 1 — Supplementary Information [file 42004_2021_456_MOESM1_ESM.pdf]

Supplementary Information

Rotaxane rings promote oblique packing and extended lifetimes in DNA-templated molecular dye aggregates

Matthew S. Barclay,[1] Simon K. Roy,[1] Jonathan S. Huff,[1] Olga A. Mass,[1] Daniel B. Turner,[1] Christopher K. Wilson,[1] Donald L. Kellis,[1] Ewald A. Terpetschnig,[2] Jeunghoon Lee,[1,3] Paul H. Davis,[1] Bernard Yurke,[1,4] William B. Knowlton,[1,4] Ryan D. Pensack[1,]*

[1]Micron School of Materials Science & Engineering, [3]Department of Chemistry & Biochemistry, and [4]Department of Electrical & Computer Engineering, Boise State University, Boise, Idaho 83725, USA

[2]SETA BioMedicals, Urbana, Illinois 61801, USA

Corresponding author(s):

*ryanpensack@boisestate.edu

Note	Contents	Page
1	Covalent attachment of commercial squaraine and squaraine:rotaxane dyes to DNA backbone	S-3
2	DNA Holliday junctions adopt a stacked conformation	S-5
3	Squaraine and squaraine:rotaxane monomer photophysics are largely similar for dyes located on different parts of the Holliday junction	S-7
4	Theoretical modeling evidences H-packing in the squaraine dimer and oblique packing in the squaraine:rotaxane dimer	S-9
5	Gaussian fitting identifies four components within the absorption spectrum of the squaraine dimer solution	S-12
6	Differences in extinction coefficient upon aggregation	S-14
7	Vibronic and electronic character in excitonic transitions of the squaraine dimer	S-15
8	Electronic coherence is maximized when two excitonically split transitions are of equal intensity	S-16

9	Only a narrow range of oblique angles are able to reproduce the excitonic transition intensities of the oblique aggregate absorption spectrum	S-19
10	Supplementary methods	S-22
11	Squaraine and squaraine:rotaxane monomer lifetimes measured via time-correlated single photon counting	S-24
12	Fluorescence emission in dimer solutions comes primarily from monomer species	S-25
13	Fluorescence emission quenching in squaraine and squaraine:rotaxane dimer solutions	S-26
14	Femtosecond transient absorption measurements observe extended lifetimes in squaraine:rotaxane dimer solution	S-27
15	Four- and three-component kinetic schemes best model the squaraine and squaraine:rotaxane dimer solutions, respectively	S-36
16	Transient absorption of squaraine:rotaxane dimer solution is largely invariant to pump wavelength	S-45
17	Rotaxane ring remains attached to squaraine dye after attachment to DNA and of the nanostructure in aqueous solution	S-48
18	Characterization of the temporal pulsewidth of the pump beam in the transient absorption measurement	S-51
19	Spectrum of the white-light continuum used as the probe beam in the transient absorption measurement	S-52
	Supplementary references	S-53

<u>Supplementary Note 1</u>: Covalent attachment of commercial squaraine and squaraine:rotaxane dyes to DNA backbone

Squaraine and squaraine:rotaxane dyes, Square-660-NHS ester (K8-1352, SETA BioMedicals) and SeTau-670-NHS ester (K9-4169, SETA BioMedicals), were attached to DNA via reaction of the *N*-hydroxysuccinimide (NHS) ester group of the dye with the amino group of the non-nucleosidic serinol linker (**Supplementary Figure 1**). The attachment and subsequent HPLC purification were carried out by Bio-Synthesis.

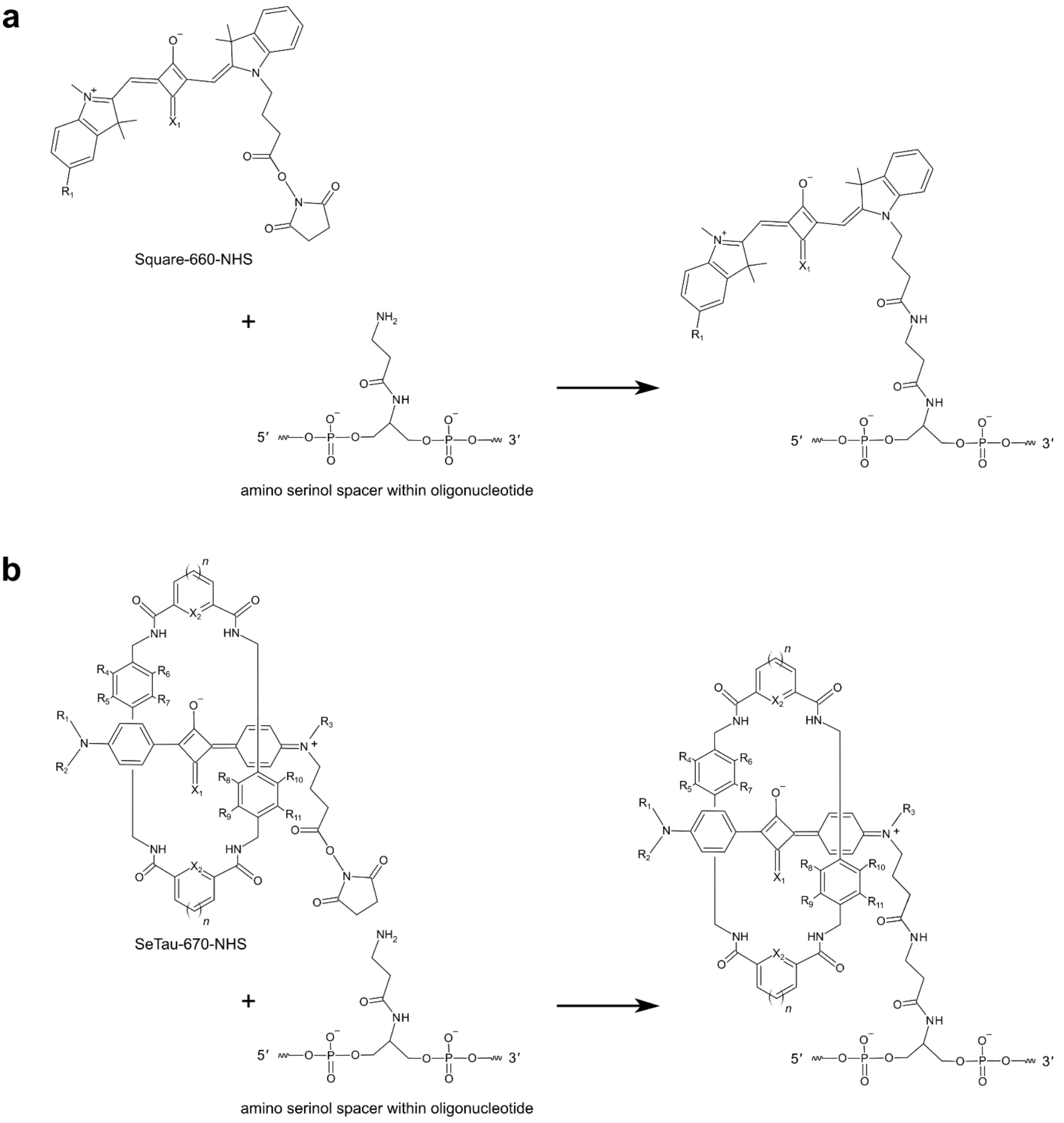

Supplementary Figure 1. Post-modification of oligonucleotide containing a serinol linker with **a** Square-660-NHS (K8-1352) and **b** SeTau-670-NHS (K9-4169). Post-modification and purification were carried out by Bio-Synthesis and the dyes were supplied by SETA BioMedicals. The exact chemical structures of the Square-660-NHS and SeTau-670-NHS dyes are proprietary.

Supplementary Note 2: DNA Holliday junctions adopt a stacked conformation

In order to identify the conformation adopted by the DNA Holliday junctions, we performed thermal denaturation experiments. Holliday junctions generally form either a stacked antiparallel conformation or an open conformation, depending on whether the arms of the four-way junction are oriented coaxially [2,3]. Due to the additional stability provided by the coaxial base pairing, the thermal unfolding of the stacked conformation occurs in one transition step and requires a higher temperature than that of the open conformation, which proceeds in a series of transitions.

As has been described in previous work by Mass *et al.* [4], and the melting profiles were measured using a Varian Cary5000 spectrophotometer equipped with a thermal probe (Agilent Technologies Cary Temperature Controller G9808), which monitored the absorption at 260 nm as the temperature was increased from 25 °C to 95 °C. Mass *et al.*'s thermal denaturation experiments previously determined that the unlabeled DNA HJs and the HJs templated with Square-660-NHS (SQ) in the form of the dimer that we study in this manuscript both adopt the stacked conformation in solutions of 1×TBE + 15 mM $MgCl_2$ running buffer. The stacked conformers exhibited a single denaturation transition at a melting temperature of ca. 60 °C. The melting profile for the unlabeled DNA HJs and the DNA HJs templated with SeTau-670-NHS (SR) in the form of a dimer, each using the same running buffer 1×TBE + 15 mM $MgCl_2$, are provided in **Supplementary Figure 2**. The melting temperatures were determined by fitting a Gaussian curve to the numerical first derivative of the melting profile. The result in **Supplementary Figure 2** demonstrates that the DNA HJ of the SR dimer solution exhibits a single denaturation transition at ca. 59 °C, indicating that it adopts the stacked conformation.

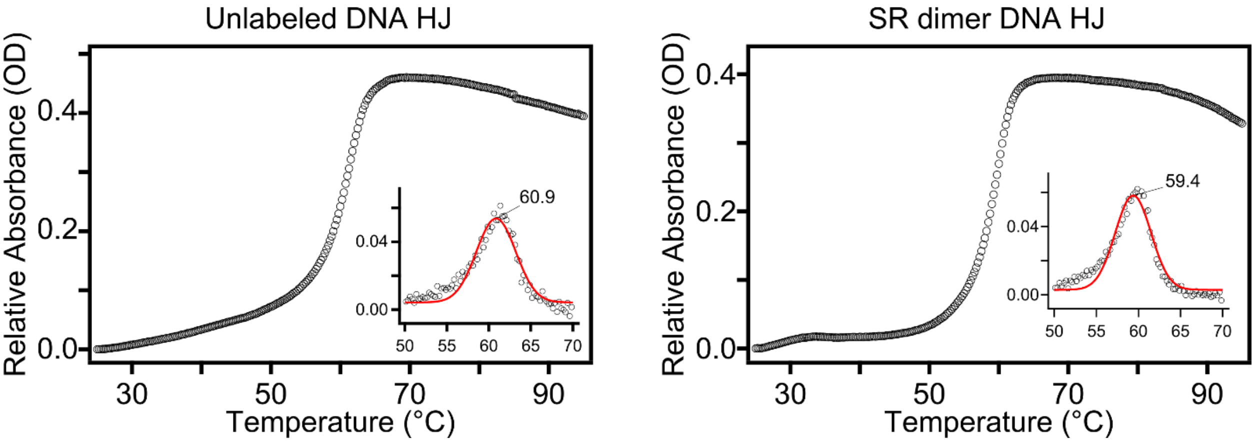

Supplementary Figure 2. Melting temperatures of unlabeled DNA Holliday junctions and DNA Holliday junctions templated with SR dyes in the form of a dimer. Thermal denaturation was performed for both samples in 1×TBE + 15 mM $MgCl_2$ solution, containing 1.5 μM and 3.0 μM DNA construct for the unlabeled and SR dimer HJ solutions, respectively. Insets display the first derivative of the melting curve as a function of temperature and are fitted with a Gaussian curve (red) to determine the central melting temperature.

<u>Supplementary Note 3</u>: Squaraine and squaraine:rotaxane monomer photophysics are largely similar for dyes located on different parts of the Holliday junction

Here, we show that the squaraine (SQ) and squaraine:rotaxane (SR) dyes templated as monomers in DNA Holliday junctions (HJs) exhibit similar photophysics, regardless of which DNA strand the dye is attached to. We prepared monomer solutions of HJs by templating the corresponding SQ or SR dye into either of the DNA sequences in **Supplementary Table 1** (which are also the two dye-labeled strands used to form the transverse dimer aggregate solutions) and adding equimolar amounts of the unlabeled forms of the three other DNA strands, which are shown in **Fig. 2** of the main text.

Supplementary Table 1. Dye-labeled DNA strands used to form Holliday junctions.

Strand Name	Sequence
A	5'-ATATAATCGCTCG/**dye**/CATATTATGACTG-3'
C	5'-CACTCACATTCCA/**dye**/CTCAACACCACAA-3'

Steady-state spectroscopic measurements of absorption and fluorescence emission for the "A"- and "C"-labeled monomers for each dye are shown in **Supplementary Figure 3**. When normalized, the spectra are nearly identical, which indicates negligible discrepancies between the steady-state photophysics for the monomer dyes templated at different strand positions in the HJ. Additionally, the fluorescence quantum yield (FQYs) for each of these samples were determined and are given in **Supplementary Table 2**.

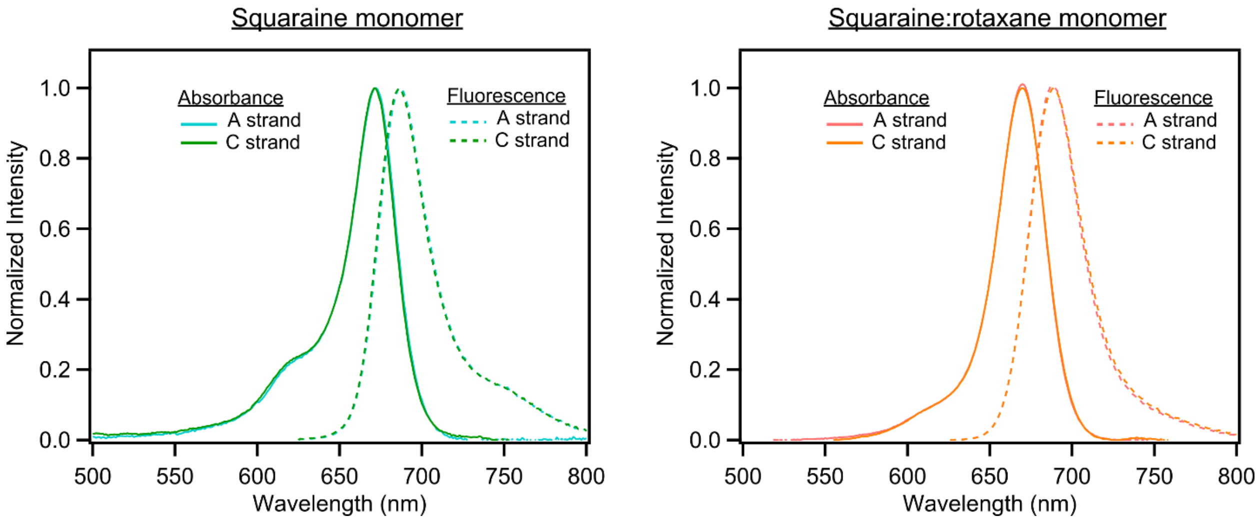

Supplementary Figure 3. Steady-state absorption and fluorescence emission spectra of squaraine (SQ) and squaraine:rotaxane (SR) monomers templated on the "A" or "C" strands in a DNA Holliday junction.

Supplementary Table 2. Fluorescence quantum yields (FQY) measured for squaraine (SQ) and squaraine:rotaxane (SR) monomer solutions.

Dye	Strand	FQY
SQ	A	0.37 $\pm$ 0.05
	C	0.36 $\pm$ 0.02
SR	A	0.49 $\pm$ 0.02
	C	0.48 $\pm$ 0.04

As in the steady-state absorption spectra, the FQY values for each dye monomer solution exhibit very little discrepancy between the different labeled strands. Therefore, we focus our discussions on dye monomers labeled onto only one strand of the HJ. All subsequent references to the "monomer" solutions will be constrained to dyes templated onto the "C" strand, unless otherwise noted.

<u>Supplementary Note 4</u>: **Theoretical modeling evidences H-packing in the squaraine dimer and oblique packing in the squaraine:rotaxane dimer**

Here, we present the polar and Cartesian coordinates along with three-dimensional results for the theoretical modeling of the transition dipole moments (TDMs) of the squaraine (SQ) and squaraine:rotaxane (SR) dimer solutions based on the Kühn-Renger-May (KRM) model [5–8]. The input parameters used in each of the calculations are listed in **Supplementary Table 3**, including the inputs obtained from the experimental absorption spectrum of the monomer in order to calculate the characteristic exciton hopping parameter (J_0) that is used as an input in the KRM modeling of the dimer, which has been previously described in detail by Mass *et al.* [4]. The calculated outputs of these parameters are shown in **Supplementary Tables 4** and **7**, and the positions of the aggregated TDM centers and the orientations of the dye TDMs via zenith (θ_i) and azimuthal (φ_i) angles are given in **Supplementary Tables 5** and **6**.

Supplementary Table 3. Input fitting parameters for the KRM calculations of SQ and SR monomers and aggregates.

	Monomer	
	**SQ**	**SR**
Energy of vibron (E_v), eV	0.153	0.160
Displacement of excited state vibronic potential (*d*), dimensionless units	0.68	0.46
Energy loss damping constant (Γ), eV	0.033	0.037
	**Dimer**	
	**SQ**	**SR**
Characteristic exciton hopping parameter (J_0^*), eV·nm^3	0.044	0.060
Energy offset from monomer (E_{of}), eV	0.00	0.01
Energy of vibron (E_v), eV	0.153	0.160
Displacement of excited state vibronic potential (*d*), dimensionless units	0.68	0.46
Energy loss damping constant (Γ), eV	0.033	0.037
Length of transition dipole moment, nm	1.3	1.3
Closest distance between the long axes of any pair of dyes, nm	0.340	0.345

Supplementary Table 4. Output parameters extracted from KRM calculations of SQ and SR monomers.

	Monomer	
	SQ	SR
Dipole moment (M_{01}), Debye	11.17	13.07
Characteristic exciton hopping parameter (J_0^*), eV·nm^3	0.044	0.060

Supplementary Table 5. KRM fitting outputs describing the orientation and positions of the dye TDMs in the squaraine dimer.

Dye	θ_i (°)	φ_i (°)	x_i (nm)	y_i (nm)	z_i (nm)
1	92.20	-9.67	1.37	-2.46	0.80
2	88.95	12.80	1.30	-1.93	0.60

Supplementary Table 6. KRM fitting outputs describing the orientation and positions of the dye TDMs in the squaraine:rotaxane dimer.

Dye	θ_i (°)	φ_i (°)	x_i (nm)	y_i (nm)	z_i (nm)
1	61.62	-38.70	1.62	1.31	1.71
2	117.30	28.49	1.97	2.08	0.79

Supplementary Table 7. Output parameters extracted from KRM calculations of SQ and SR dimer aggregates.

	Dimer	
	SQ	SR
Excitonic hopping parameter ($J_{1,2}$), meV	66.2	50.8
Calculated closest distance between transition dipole moments (d_{min}), nm	0.340	0.349

Center-to-center distance (*R*), nm	0.5681	1.247
Slip angle (θ_s)	75°	60°
Oblique angle (α)	22.7°	85.1°

The results from the main text (**Fig. 3c,f**) are shown from the three axial planes (XY, YZ, and XZ) for ease of visualization below. **Supplementary Figure 4** shows the TDMs obtained from the KRM modeling of the SQ dimer solution, and **Supplementary Figure 5** displays the corresponding results for the SR dimer solution.

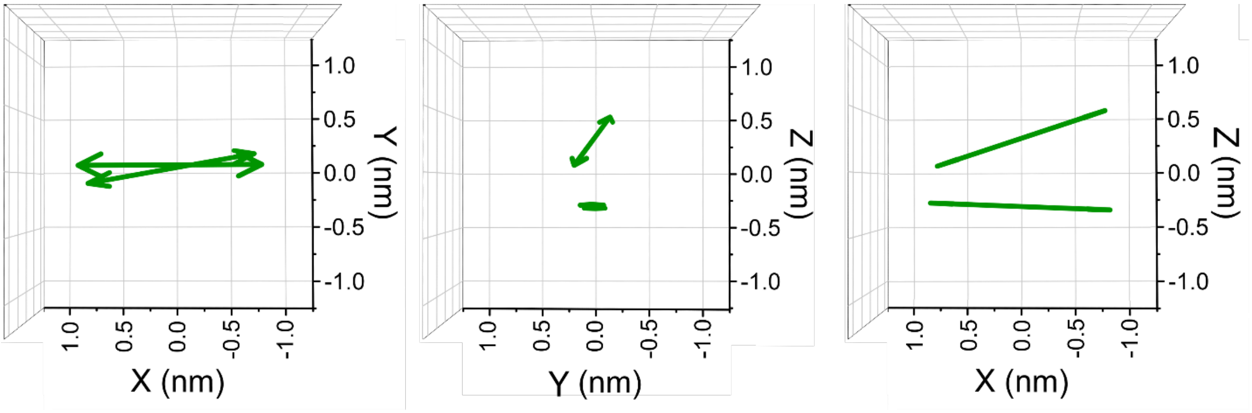

Supplementary Figure 4. Transition dipole moments (TDMs) derived from the KRM modeling of the absorption and CD spectra of the SQ dimer solution. The TDMs corresponding to the two dyes in the dimer structure are shown as green, double-headed arrows, and projected along the XY, YZ, and XZ planes.

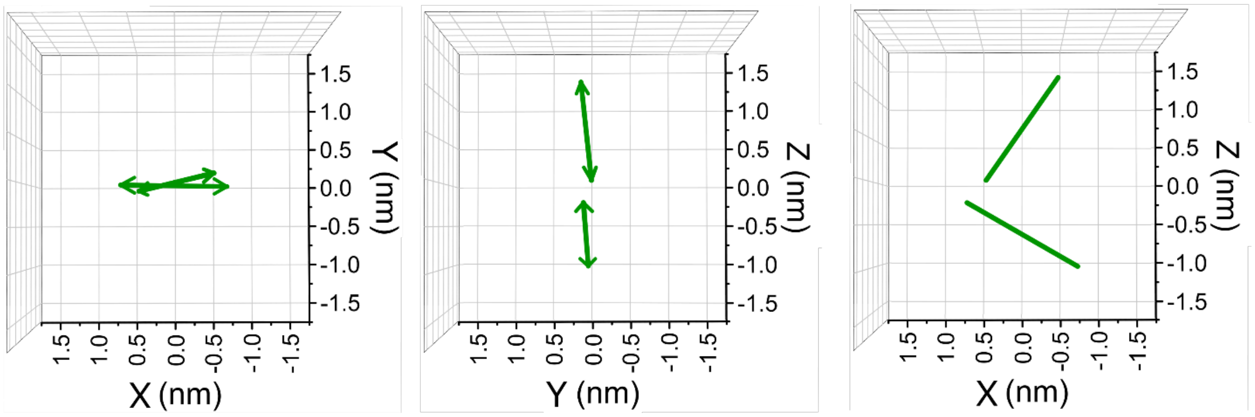

Supplementary Figure 5. Transition dipole moments (TDMs) derived from the KRM modeling of the absorption and CD spectra of the SR dimer solution. The TDMs corresponding to the two dyes in the dimer structure are shown as green, double-headed arrows, and projected along the XY, YZ, and XZ planes.

<u>Supplementary Note 5</u>: Gaussian fitting identifies four components within the absorption spectrum of the squaraine dimer solution

The steady-state absorption spectrum of the squaraine (SQ) dimer solution was fit with multiple Gaussian functions in order to better identify the absorption bands underlying the absorption spectrum. Based on the procedure described by Mass *et al.* [4], the absorption spectrum of the SQ dimer solution was fit with four Gaussian functions resulting in the plot shown in **Supplementary Figure 6**. The fit parameters for each component are listed in **Supplementary Table 8**, where, for each Gaussian function (A_n), x_c is the peak wavelength, w is the standard deviation, and FWHM is the full-width at half-maximum.

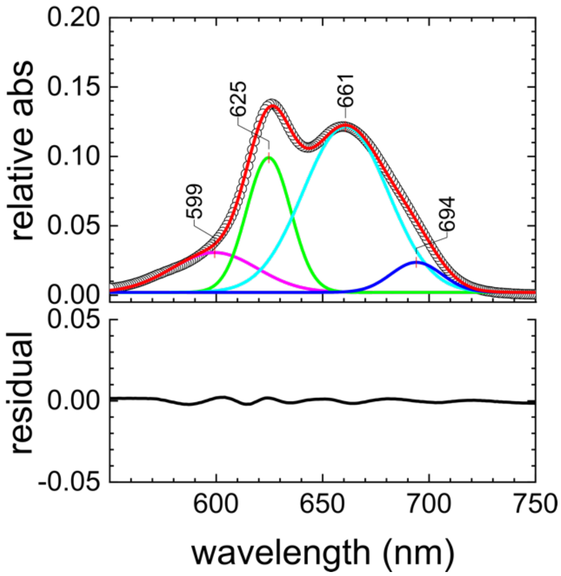

Supplementary Figure 6. The steady-state absorption spectrum of the squaraine (SQ) dimer solution fit with four Gaussian functions. The experimental absorption spectrum (open black circles) is included, along with the cumulative fit (solid red line), which is obtained by summing the four Gaussian functions shown. The residuals between the experimental spectrum and cumulative fit is shown on the bottom.

Supplementary Table 8. Gaussian fitting parameters of SQ660 transverse dimer absorption spectrum.

Spectral Band	x_c (nm)	w (nm)	FWHM (nm)	Area	Adjusted R^2
A_1	694	24	28	0.65	0.999
A_2	661	40	47	5.99	0.999
A_3	625	21	25	2.54	0.999
A_4	599	40	47	1.44	0.999

<u>Supplementary Note 6</u>: **Differences in extinction coefficient upon aggregation**

Here we plot the steady-state absorption spectra in units of $M^{-1}cm^{-1}$ to better compare the effect of aggregation on the extinction coefficients in the SQ and SR dimer solutions, shown in **Supplementary Figure 7**. Upon aggregation, the peak extinction coefficient of the SQ dimer is not significantly different from that of the monomer. Alternatively, the peak extinction coefficient of the SR dimer is ca. 39% larger than that of the monomer, which may indicate the presence of overlapping excitonic transitions.

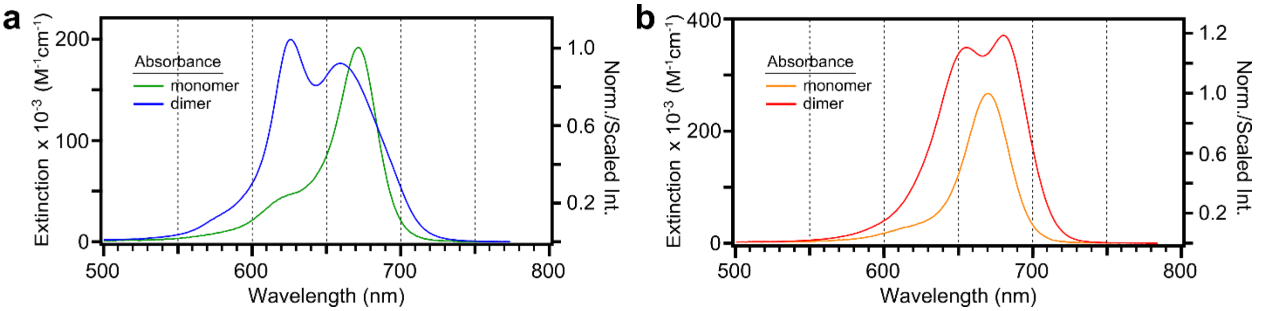

Supplementary Figure 7. Steady-state absorption spectra for the **a** SQ and **b** SR dimer solutions.

<u>Supplementary Note 7</u>: Vibronic and electronic character in excitonic transitions of the squaraine dimer

Here, we provide an analysis of the pure electronic and vibronic contributions of the excitonic transitions in the SQ dimer for comparison with the results for the SR dimer in **Fig. 4a,b** in the main text. The fits from the KRM modeling, which assume that the SQ dimer has a homogeneous population, are shown in **Supplementary Figure 8**. The results indicate that, as observed for the SR dimer and expected for excitonic coupling, the two lowest-energy transitions are of primarily electronic origin (ca. 98% and 82%). However, in contrast to the near-equal oscillator strengths of the excitonic transitions in the SR dimer, the SQ dimer exhibits a dominant electronic transition intensity at 660 nm and a much weaker electronic transition at 694 nm. The discrepancy in the simulated relative intensities of these two transitions suggests that electronic coherences prepared in the SQ dimer would have significantly weaker amplitude than those of the SR dimer.

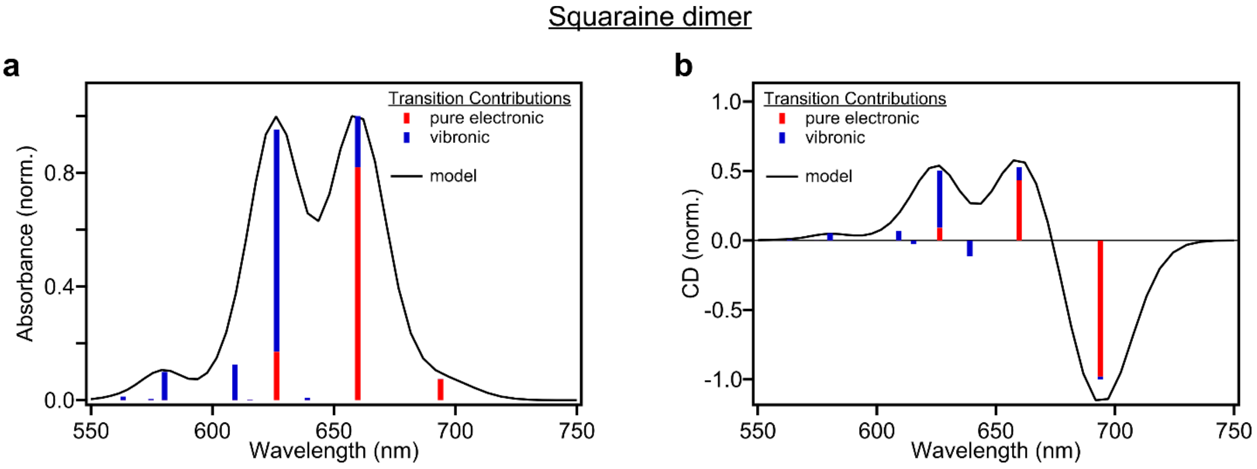

Supplementary Figure 8. Electronic and vibronic contributions to the **a** steady-state absorption and **b** CD spectra of the SQ dimer structure.

However, as observed in **Fig. 5a** and **Supplementary Note 14**, the population of the SQ dimer solution is structurally heterogeneous, which complicates the interpretation of the simulated spectra. We make a point to clarify that, because the currently implemented KRM model assumes a single population of aggregates, therefore the above interpretations largely inconclusive.

<u>Supplementary Note 8</u>: Electronic coherence is maximized when two excitonically split transitions are of equal intensity

Here, we present a short mathematical justification illustrating that equal intensity transitions are ideal for generating the largest possible electronic coherence signal in a time-resolved spectroscopy measurement of molecular excitons. We describe electronic coherence using the ubiquitous three-state model in **Supplementary Figure 9**. The schematic in panel **a** displays the energy levels and transitions of the three-state system, including the ground electronic state, |g⟩, and two excitonically coupled electronic states of lower and higher energy (|1⟩ and |2⟩, respectively). The transition dipoles between the ground and excited states are generically represented by μ_1 and μ_2 for transitions between |g⟩↔|1⟩ and |g⟩↔|2⟩, respectively. **Supplementary Figure 9b** shows double-sided Feynman diagrams [9], which represent the response of the molecular system to a series of light-matter interactions. The diagrams describe two representative mechanisms that involve the generation of an electronic coherence between the excitonically coupled states in this three-state model for the non-rephasing pulse sequence. The analysis is identical for the rephasing pulse sequence. The left and right vertical lines in each diagram express the time-evolution of the *ket* and *bra* of the density matrix, respectively [10]. Time increases vertically starting from the bottom, where the population of the system begins in the ground electronic state, represented in the density matrix as |g⟩⟨g|. Each light-matter interaction is represented as an arrow, indicating a perturbation of the density matrix that results in a transition of the *bra* or *ket* state. Arrows pointing toward or away from the vertical lines correspond to (stimulated) absorption or stimulated emission transitions, respectively; the final arrow, depicted as a wave-like line, indicates that the final interaction is the emission of light; therefore it always points away from the vertical lines. The state of the system after the final interaction is |g⟩⟨g| in both diagrams.

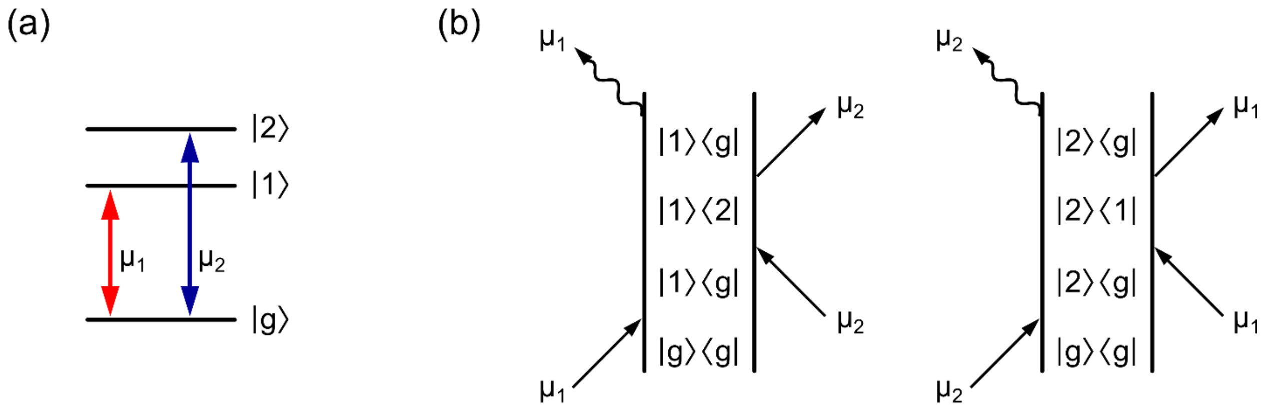

Supplementary Figure 9. Energy level schematic and Feynman diagrams for a three-state model of electronic coherence. (**a**) Energy level schematic of the three-state system, where |g⟩ is the ground electronic state and |1⟩ and |2⟩ are the low-lying and high-lying excitonically coupled states, respectively. Electronic transitions between |g⟩ and |1⟩ are represented by μ_1, and transitions between |g⟩ and |2⟩ are represented by μ_2. (**b**) Representative double-sided Feynman diagrams for non-rephasing pathways of electronic coherences created between states |1⟩ and |2⟩.

Following the diagram on the left of **Supplementary Figure 9b** as an example, the first light-matter interaction (μ_1) occurs at a time we define as τ_1, and is a transition of the *ket* component from |g⟩ to |1⟩. This transition generates a coherence between the ground and lowest-lying excitonic state, |1⟩⟨g|, which oscillates with a frequency of ω_{1g} that is the difference frequency between the two states. The |1⟩⟨g| coherence propagates until a time τ_2, when the second transition (μ_2) excites the *bra* component to form the electronic coherence between the excitonic states, |1⟩⟨2|, which oscillates with a frequency of ω_{12}. The third perturbative interaction (μ_2) takes place at τ_3 and de-excites the *bra* from ⟨2| to ⟨g|. The final response of the system to these three perturbative light-matter interactions is to emit a photon, which causes the *ket* to relax back down to the ground state. Based on these interactions, the third-order molecular response function, $R^{(3)}$, is described by **Supplementary Equation 1**, and directly relates to the amplitude of the quantum-beat signal observed in a time-resolved spectroscopy measurement [10].

$$R^{(3)}(\tau_1,\tau_2,\tau_3) \propto |\mu_1|^2|\mu_2|^2\, e^{-i\omega_{1g}\tau_1}\, e^{-i\omega_{12}\tau_2}\, e^{-i\omega_{1g}\tau_3} \qquad (1)$$

Therefore, the amplitude of the oscillations, A, is directly proportional to the product of transition probabilities for each excitonic state, $|\mu_1|^2|\mu_2|^2$, which is directly proportional to the product of oscillator strengths for each electronic transition, $f_1 f_2$.

$$A\,(f_1,f_2) \propto f_1 \cdot f_2 \qquad (2)$$

Since our goal is to determine which combination of f_1 and f_2 values maximizes the amplitude of the oscillations due to electronic coherence, we assume that the total oscillator strength remains constant (**Supplementary Equation 3**).

$$f_1 + f_2 = c \tag{3}$$

Accordingly, the maximum amplitude is found by setting the derivative with respect to either term equal to zero:

$$\begin{aligned} 0 &= \frac{\mathrm{d}}{\mathrm{d}f_1}\left[A\left(f_1, f_2\right)\right] \\ &= \frac{\mathrm{d}}{\mathrm{d}f_1}\left[f_1 \cdot f_2\right] \\ &= \frac{\mathrm{d}}{\mathrm{d}f_1}\left[f_1 \cdot \left(c - f_1\right)\right] \\ &= c - 2f_1 \end{aligned} \tag{4}$$

From **Supplementary Equation 4**, it is straightforward to determine that the amplitude of the coherent oscillation is at a maximum when each oscillator strength is equal to half the value of c. Thus, the largest possible electronic coherence is observed when the excitonic transitions are of equal intensity.

Supplementary Note 9: Only a narrow range of oblique angles are able to reproduce the excitonic transition intensities of the oblique aggregate absorption spectrum

In this supporting section, we report the details and assumptions that were implemented in the model used to investigate the effect of the oblique angle, α, on the absorption spectrum of a system composed of two transition dipole moments (TDMs), as well as the narrow range of oblique angles that are able to reproduce the near equal excitonically split transition intensities observed for the steady-state absorption spectrum of the squaraine:rotaxane (SR) dimer solution. Given the promising results obtained by the Kühn-Renger-May (KRM) model for the SR dimer (**Fig. 3**), our investigation of the oblique angle dependence of the simulated absorption spectra utilizes the steady-state absorption spectrum of the SR monomer and keeping all coupling parameters constant. **Supplementary Figure 10** shows a more detailed schematic of the model presented in the main text (**Fig. 4**). Each dye is assumed to have a length of 15 Å, where, to account for physical bulk, the length of the TDM is assumed to be 13 Å. The TDMs are restricted to be in the XZ plane, and one dye is fixed to be horizontal along the X axis. The other dye is fixed at one end, such that the distance between dyes also remains fixed at d_{min} = 3.45 Å, which was chosen to approximately correspond to the stacking distance between neighboring bases [11]. The oblique angle is defined based on the dot product between the unit vectors, $\overrightarrow{n_1}$ and $\overrightarrow{n_2}$, for each dye (**Supplementary Equation 5**).

$$\alpha = cos^{-1}(\overrightarrow{n_1} \cdot \overrightarrow{n_2}) \tag{5}$$

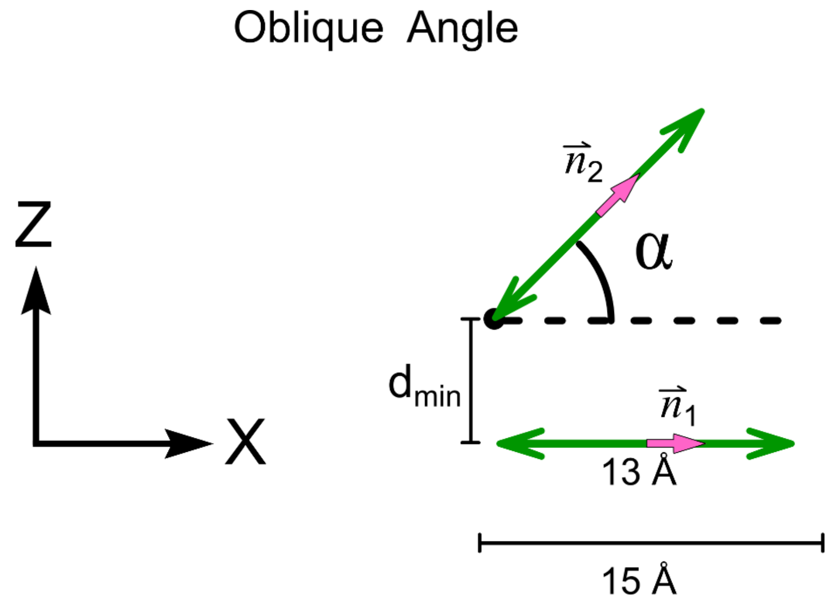

Supplementary Figure 10. Schematic showing the oblique angle between two molecules in a dimer that was varied to produce the absorption spectra shown in **Fig. 4** and **Supplementary Figure 11**. The transition dipole moments (TDMs) of the molecules are represented with green arrows and are assumed to be oriented along the long axis of the molecule. The unit vectors of each TDM are shown as pink arrows pointing outward from the midpoint. The oblique angle is varied by rotating the TDM around the point indicated by the black circle, keeping one end of the dipole fixed at that point, and a fixed distance, d_{min}, between the TDMs. The dashed line corresponds to a relative orientation between molecules where $\alpha = 0°$.

As we alter the oblique angle around the axis of rotation, the center-to-center distance (R) and slip angle (θ_s) defined in previous work [4] are altered correspondingly, as shown in **Supplementary Table 9** for all the oblique angles reported in this manuscript.

Supplementary Table 9. Geometric parameters [4] that result from varying the oblique angle, α.

**Oblique Angle (α)**	**Slip Angle (θ_s)**	**Center-to-center distance (R), nm**
0°	90.00°	0.345
40°	78.02°	0.845
65°	67.09°	1.112
75°	62.54°	1.205
85°	57.92°	1.289
95°	53.26°	1.363
105°	48.56°	1.427
110°	46.21°	1.454
180°	12.95°	1.539

Using this model for varying the oblique angle, the near equal intensity excitonic transitions that are characteristic of the oblique packing arrangement are observed to occur within a limited range of angles. **Supplementary Figure 11** illustrates the narrowness of this range by plotting the results for oblique angles between 65° and 105°. We observe that the peak intensities of the excitonic bands differ by nearly 20% at 75° and 95°, suggesting that a shift of more than ±10° may result in significant changes to the relative intensities of the excitonic transitions.

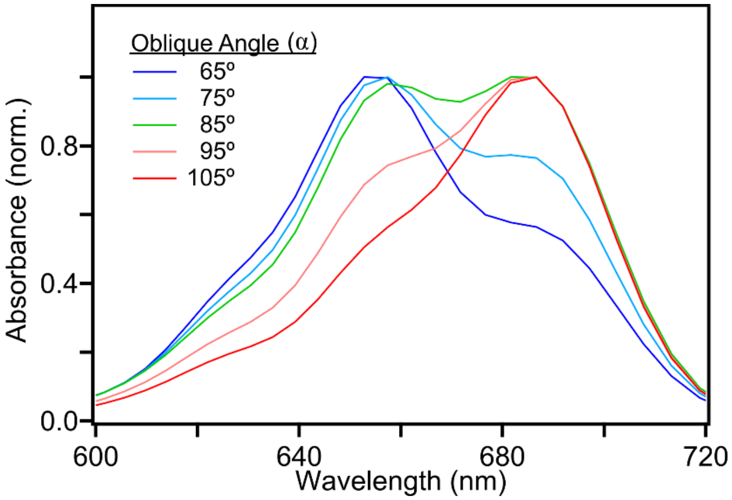

Supplementary Figure 11. Simulated absorption spectra as a function of oblique angle, α. Dipoles are assumed to be coplanar with a fixed distance of nearest approach, modeled using parameters determined from the experimental data of the SR monomer solution.

<u>Supplementary Note 10</u>: Supplementary methods

<u>Polyacrylamide gel electrophoresis (PAGE)</u>

Prior to measurements of fluorescence emission and excitation, the Square 660 and SeTau 670 dimer solutions were subjected to non-denaturing polyacrylamide gel electrophoresis (PAGE) to isolate the Holliday junction dye dimer strands. The non-denaturing 5% polyacrylamide gels, 1.5 mm thick, were cast in 1×TBE + 15 mM $MgCl_2$ buffer. Each DNA construct was diluted in a loading buffer (20% v/v Ficoll [Sigma-Aldrich, St. Louis, MO] and 20% v/v bromophenol blue [Sigma-Aldrich, St. Louis, MO]) to a final concentration of 7 μM in 45 μL. The samples were loaded onto the gel (15 μL per lane) in parallel. The electrophoresis was run at a constant voltage of 150 V and constant temperature of 17 °C in a 1×TBE + 15 mM $MgCl_2$ running buffer. The resultant gel was analyzed with a FluorChem Q imager (Alpha Innotech, San Leandro, CA) using the ex. 632 nm / em. 691 nm channel. Separated bands corresponding to ssDNA (i.e., non-hybridized oligos), dimers, and higher order DNA constructs were observed, as shown in **Supplementary Figure 12**. The dimer construct bands were excised, crushed, placed in Freeze 'N Squeeze spin columns (Bio-Rad, Hercules, CA), and subjected to 3 squeeze-freeze cycles according to the manufacturer's instructions to afford purified DNA Holliday junction templated Square 660 and SeTau 670 dimers in 1×TBE + 15 mM $MgCl_2$.

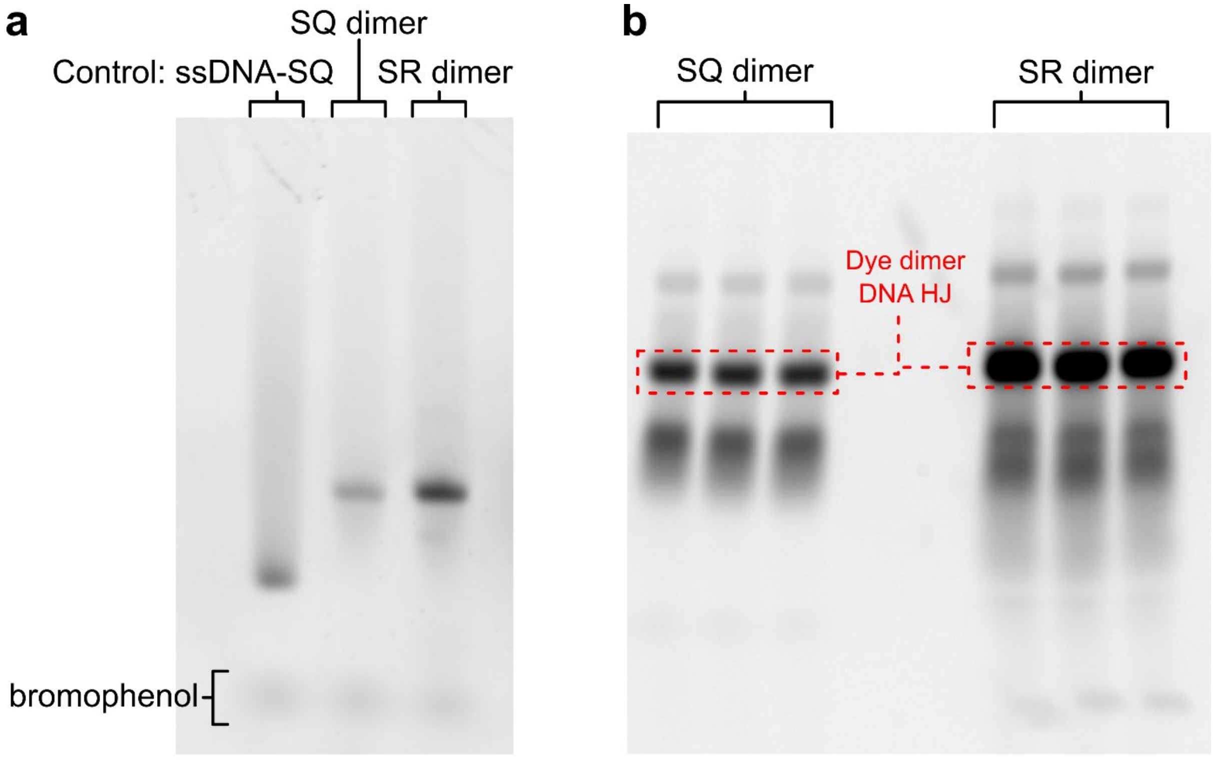

Supplementary Figure 12. Fluorescent images of 5% nondenaturing PAGE 1.5 mm run at 150 V, 17 °C in 1×TBE. **a** Analytical PAGE gel loaded with 0.15 µmol ssDNA-SQ, 0.24 µmol SQ dimer DNA HJ, and 0.24 µmol SR dimer DNA HJ. **b** Purification PAGE gel loaded in 3 lanes with SQ dimer DNA HJ and in 3 lanes with SR dimer DNA HJ, loading 115 µmol per lane. Bands corresponding to the dye dimer DNA HJs were excised and subjected to dye extraction.

Fluorescence Excitation

Fluorescence excitation spectra were measured for Square 660 and SeTau 670 monomer solutions along with dimer solutions that had been PAGE purified. The measurements were obtained using a Varian Cary Eclipse fluorescence spectrophotometer (Agilent Technologies, Santa Clara, CA). Samples were contained in a 1 cm pathlength quartz cuvette (Starna Cells, Atascadero, CA), and the detection wavelength was set to 740 nm for each solution.

Time-Correlated Single Photon Counting

Fluorescence lifetimes of the Square 660 and SeTau 670 monomer solutions were measured using a PicoQuant FluoTime 250 time-correlated single photon counting (TCSPC) spectrometer (PicoQuant, Berlin, Germany). The samples were contained within a 1 cm path length quartz cuvette (Starna Cells, Inc., Atascadero, CA), and diluted to ensure the optical density of the solution was less than 0.05 within the spectral range between 500 and 800 nm. The excitation and emission detection wavelengths for each monomer solution were 650 and 750 nm, respectively, and were chosen to avoid overlap with emission and absorption bands, respectively. The instrument response function (IRF) was measured using a solution of Ludox colloidal silica (PicoQuant, Berlin, Germany), from which the time-resolution of the spectrometer was determined based on the ca. 80 ps full-width at half-maximum (FWHM) of the IRF.

<u>Supplementary Note 11</u>: Squaraine and squaraine:rotaxane monomer lifetimes measured via time-correlated single photon counting

We determine the excited-state lifetimes of the squaraine (SQ) and squaraine:rotaxane (SR) monomer solutions by measuring the decay of the fluorescence emission via time-correlated single photon counting (TCSPC). The results of the TCSPC measurements are shown in **Supplementary Figure 13**, and the fluorescence lifetimes are determined by fitting the data to a single exponential that includes the instrument response function (IRF). Based on the fits to the TCSPC data, the lifetimes of the SQ and SR monomer solutions are ca. 2.88 and 2.85 ns, respectively.

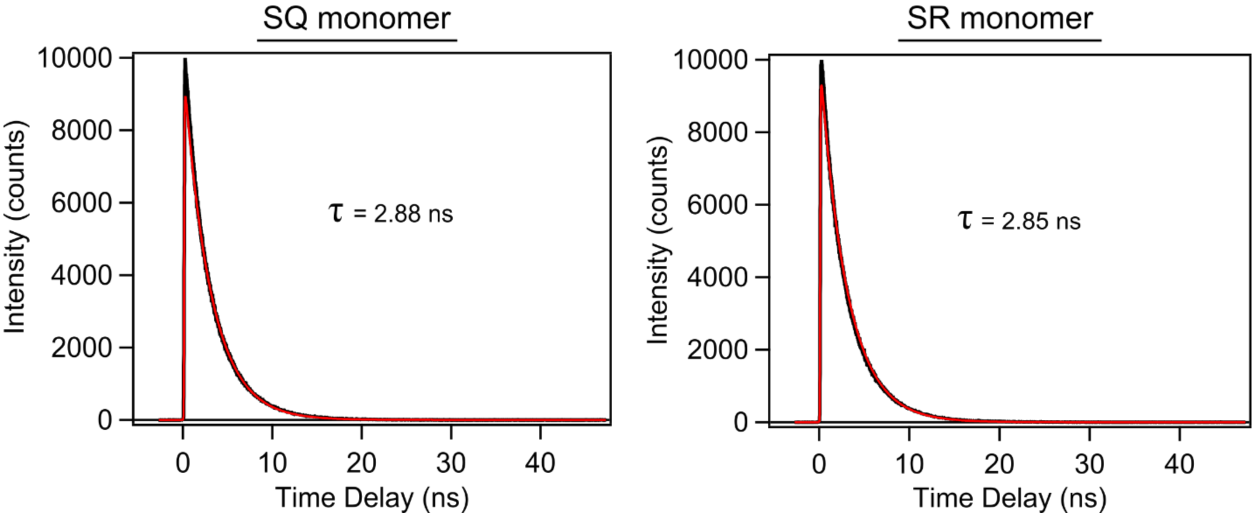

Supplementary Figure 13. Time-correlated single photon counting (TCSPC) measurements of squaraine (SQ) and squaraine:rotaxane (SR) monomers templated using a DNA Holliday junction. For each solution, the measurement used an excitation wavelength of 620 nm and the fluorescence emission intensity was observed at 750 nm.

<u>Supplementary Note 12</u>: Fluorescence emission in dimer solutions comes primarily from monomer species

We measure fluorescence excitation profiles for the squaraine (SQ) and squaraine:rotaxane (SR) dimer solutions in order to identify the primary emissive species in each solution. The fluorescence excitation profiles are obtained by measuring the fluorescence emission intensity at a set detection wavelength and varying the excitation wavelength. The resultant fluorescence excitation spectra provide information on the identity of the emissive species based on comparisons between the fluorescence excitation profile and the steady-state absorption spectrum.

Supplementary Figure 14 shows the fluorescence excitation spectra for SQ and SR dimer solutions that have been PAGE purified, along with the steady-state absorption spectrum of each dimer solution, and the absorption spectra of the corresponding monomer solutions. We observe that, for both SQ and SR dyes, the fluorescence excitation profile of the dimer solution is nearly identical to that of the respective monomer solution, each of which closely traces the profile of the monomer steady-state absorption spectrum. The results of **Supplementary Figure 14** indicate that the fluorescence emission observed for each dimer solution comes primarily from monomer species, even after PAGE purification.

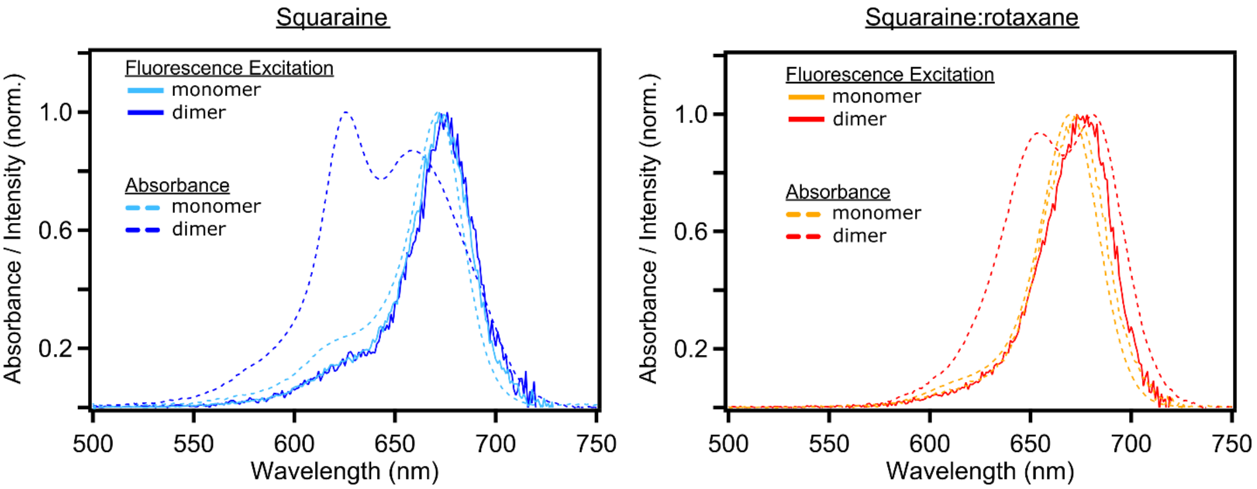

Supplementary Figure 14. Fluorescence excitation spectra of squaraine (SQ) and squaraine:rotaxane (SR) dyes templated using a DNA Holliday junction. Fluorescence excitation spectra for monomer and dimer solutions (solid lines) are observed at an emission wavelength of 740 nm, and the steady-state absorption spectra of the corresponding solutions (dashed lines) are included for comparison. All spectra are normalized to the most intense peak between 500 and 750 nm. The SQ and SR dimer solutions have been purified via PAGE.

Supplementary Note 13: Fluorescence emission quenching in squaraine and squaraine:rotaxane dimer solutions

As a preliminary survey of the excited-state dynamics of the SQ and SR dimer structures, we performed relative fluorescence emission measurements on the SQ and SR dimer solutions. Specifically, the fluorescence emission intensity of the SQ and SR dimer solutions were compared with the fluorescence emission intensity of the respective monomer solutions. **Fig. 2b** and **Fig. 2c** in the main text show that significant fluorescence emission quenching is observed in both SQ and SR dimer solutions. Similar quenching has recently been observed in aggregates of DNA-templated cyanine dyes [5,6,12,13], which was found to arise from new non-radiative decay pathways introduced upon aggregation [12,13]. Promisingly, the extent of fluorescence emission quenching appears to be weaker in the SR dimer solution compared with the SQ dimer solution. Specifically, the measured FQY values for the SQ and SR dimer solutions were 0.02 and 0.13, respectively. Complicating the interpretation that the fluorescence emission quenching is less extensive in the SR dimer solutions, however, is the observation that the fluorescence excitation spectra of the SQ and SR dimer solutions more closely match the absorption spectra of the SQ and SR monomer solutions and not the absorption spectra of the SQ and SR dimer solution (**Supplementary Note 12**). These results indicate that monomers are the primary emissive species in the dimer solutions. Given that the dimer solutions were subjected to PAGE separation and subsequent purification (**Supplementary Note 10**), we hypothesize that the monomer sub-population may be due to DNA breathing effects [14] that distort the positions of the individual dyes enough to disrupt the excitonic coupling and, consequently, the aggregate fluorescence quenching.

Although the presence of a sub-population of monomers complicates a full quantitative analysis [13], we can take the FQY values 0.02 and 0.13 measured for the SQ and SR dimer solutions, respectively, to represent upper limits for the FQY values of the SQ and SR dimer structures. While we can conclude that both SQ and SR dimers exhibit significant fluorescence emission quenching, any interpretations of the fluorescence lifetimes and FQYs of the dimer structures are obfuscated by the monomeric sub-population. As such, it is necessary to perform direct measurements of their excited-state dynamics (see **Supplementary Notes 14-16**).

Supplementary Note 14: Femtosecond transient absorption measurements observe extended lifetimes in squaraine:rotaxane dimer solution

We begin by analyzing the transient absorption (TA) spectra of the SQ and SR monomer solutions, shown in **Supplementary Figure 15**, which are reported in units of differential transmittance (i.e., ΔT/T). For both dye solutions, the positive ΔT/T signals between 600 and 700 nm are assigned as a combination of ground-state bleach (GSB) and stimulated emission (SE) features, based on comparisons with the steady-state absorption spectra in **Supplementary Figure 3**. The TA spectra also exhibit an excited-state absorption band centered at ca. 545 and 470 nm for the SQ and SR monomer solutions, respectively.

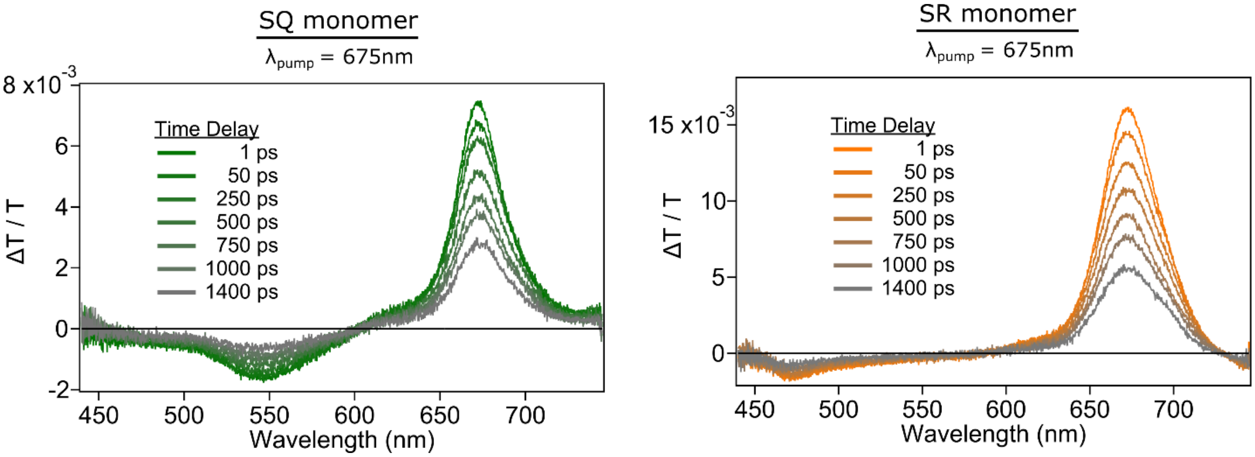

Supplementary Figure 15. Selected representative transient absorption (TA) spectra of squaraine (SQ) and squaraine:rotaxane (SR) monomers templated using a DNA Holliday junction. Spectral time delays are indicated in the legends.

Next, we analyze the TA of the SQ dimer solution. Starting with the pump wavelength tuned to the most intense absorption band at 620 nm, the results are shown in panels **a-d** of **Supplementary Figure 16**. Panel **a** displays a surface plot of the TA measurement aligned with the steady-state absorption and pump excitation profile for comparison; transient spectra are shown in panel **b**. Positive TA signals are observed between 600-700 nm, with peaks at ca. 620, 653, and 694 nm that generally align with the bands in the steady-state absorption spectrum (**Supplementary Note 5**); therefore, these signals are attributed to ground-state bleach (GSB) features. The good agreement between the steady-state absorption and positive TA bands, combined with the absence of any significant features in the lower-energy region ≥700 nm where

fluorescence emission might be expected, indicates relatively weak stimulated emission/fluorescence intensity from the dimer species, which is consistent with the substantial fluorescence quenching observed in **Fig. 2b** of the main text. The GSB features of the SQ dimer decay within about 100 ps into a weaker, longer-lived positive-going signal centered at around 680 nm that resembles the steady-state absorption of the monomer species and persists past the limit of our measured time delay (1.4 ns). The TA of the monomer-only sample (**Supplementary Figure 15**) reproduces the behavior of this weaker, longer-lived signal in the dimer solution TA measurement. Thus, we attribute this long time positive-going signal to the GSB of a small sub-population of monomers, which, as noted above, have a lifetime of ~ 2.9 ns (**Supplementary Note 11**). Negative TA signals correspond to excited-state absorption (ESA) bands, two of which are observed (near 500 nm and 540 nm) for the dimer species. The 540 nm ESA signal decays rapidly within a few ps, while the ESA at 500 nm decays synchronously with the dimer GSB. Additionally, at long time delays, a very weak ESA band is present at 550 nm, matching the TA of the monomer-only sample (**Supplementary Figure 15**). The notably shorter lifetime of the dimer GSB compared to that of the monomer demonstrates significant excited-state quenching upon aggregation, similar to the quenching observed in previous TA measurements of DNA-templated Cy5 aggregates [13].

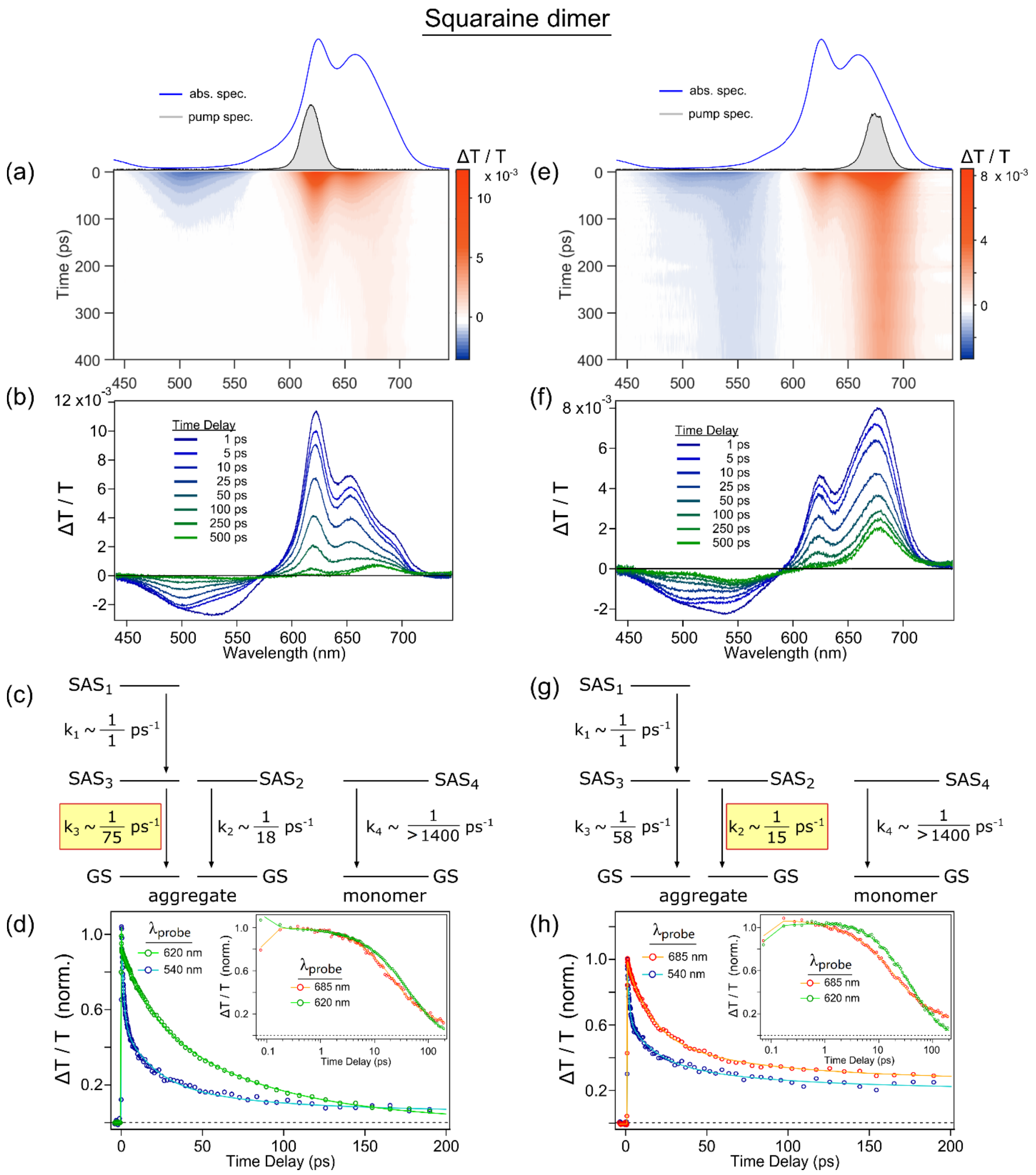

Supplementary Figure 16. Transient absorption (TA) of squaraine (SQ) dimers templated in the form of a transverse dimer using a DNA Holliday junction. (**a** and **e**) TA surface plots. Experiments were performed with incident pump wavelengths of 620 and 675 nm and pump fluences of 33 and 30 μJ cm^{-2}, respectively. Scale bar is indicated beside each plot, with the corresponding steady-state absorption and pump excitation spectra included above the surface plots. (**b** and **f**) Selected representative TA spectra. Spectral time delays are indicated in the legends. (**c** and **g**) Kinetic

schemes used to model the TA dynamics via global target analysis. Rate constants (expressed as inverse time constants) associated with conversion between different components exhibiting distinct species-associated spectra (SAS_n) are shown, with the rate constant associated with the excited-state lifetime of the SQ dimer structure preferentially excited at each pump wavelength highlighted in yellow. (**d** and **h**) TA kinetics at selected probe wavelengths (i.e., selected spectral slices of the surface plots shown in **a**, **e**). Each kinetic trace has been normalized to its maximum amplitude. Fits from the global target analysis (per the kinetic schemes shown in **c**, **g**) are overlaid on the kinetic traces. The insets in panels **d** and **h** highlight the differences between the decay traces at 620 and 685 plotted along a logarithmic time axis.

In order to quantify the excitonic lifetime of the SQ dimers, we perform global target analysis [16,17] using a four-component kinetic model. The kinetic model, which includes four components associated with three distinct structural sub-populations present in the solution, is shown in **Supplementary Figure 16c**. Details of and a full justification for the kinetic model, including the species-associated spectra (SAS) for each component, can be found in **Supplementary Note 15**. Based on the kinetic model, the global target analysis derives rate constants ($k_n = 1/\tau_n$) with corresponding time constants, $\tau_{1\text{-}4}$, of ~1, ~18, ~75, and > 1400 ps. We attribute the first and third kinetic components (associated with τ_1 and τ_3, respectively) to the primary dimer population, which exhibits a sequential decay, and assign the corresponding excitonic lifetime, highlighted in panel **c**, to the terminating τ_3 time constant of 75 ps. Evidence for these time constants is shown in **Supplementary Figure 16d**, which overlays the global fits onto normalized kinetic traces of the TA data, and shows that the ESA band at 540 nm and the primary band of the GSB (λ_{probe} = 620 nm) decay largely with the time constants of τ_1 (1 ps) and τ_3 (75 ps), respectively. In addition to the primary dimer population, we observe two other sub-populations in the SQ dimer solution when exciting at 620 nm. First, we identify a minority dimer sub-population associated with τ_2, observable in the inset of **Supplementary Figure 16d** at λ_{probe} = 685 nm. Secondly, there is a long-lived monomer sub-population that gives rise to τ_4. Thus, we find that when the SQ dimer solution is excited at 620 nm, it exhibits evidence of dimer structural heterogeneity, with a primary dimer population decaying sequentially with a 75 ps excitonic lifetime and a minority dimer population decaying mono-exponentially with a shorter 18 ps lifetime.

To further investigate the dimer structural heterogeneity identified in the global target analysis above, we subsequently performed a TA measurement on the SQ dimer solution exciting at λ_{pump} = 675 nm. We chose a pump wavelength of 675 nm because it is in close proximity to the

band associated with, and thus should selectively excite, the dimer sub-population observed at λ_{probe} = 685 nm. The results of the TA experiment conducted at this excitation wavelength are given in **Supplementary Figures 16e-16h**. When compared with the corresponding surface plot and transient spectra shown in panels **a** and **b**, the surface plot and transient spectra shown in panels **e** and **f** demonstrate a distinct pump wavelength dependence in the overall GSB signal. Furthermore, the TA spectra obtained following excitation at λ_{pump} = 675 nm do not reproduce the steady-state absorption spectrum as well as the results at λ_{pump} = 620 nm. Instead, the TA signal appears to resemble more the longer-wavelength portion of the steady-state spectrum, where we observe a broad band peaking at 660 nm. Thus, by exciting the longer-wavelength part of the steady-state absorption spectrum (with λ_{pump} = 675 nm), the aggregate sub-population that comprised the minority component of the signal observed in the λ_{pump} = 620 nm measurement is preferentially excited and becomes the primary component contributing to the TA signal. Additionally, due to the greater overlap of the monomer absorption with the λ_{pump} = 675 nm, a larger fraction of the sub-population of long-lived monomer species is excited relative to the experiment at 620 nm excitation. To quantify the finer distinctions between the TA spectra obtained with the different excitation wavelengths, we perform global target analysis utilizing the same four-component kinetic model, shown in **Supplementary Figure 16g**, which calculates aggregate time constants $\tau_{1\text{-}3}$ of ~1, ~15, and ~58 ps, as well as a monomer time constant of τ_4 > 1400 ps (see **Supplementary Note 15** for further details). The similarity of the time constants obtained at both excitation wavelengths supports the kinetic model and further validates the difference in excitonic lifetimes of the two dimer aggregate structures. These time constants are also supported by the quality of the fits to the kinetic traces in **Supplementary Figure 16h**. Additionally, the increased contribution of the more rapid ~15-18 ps decay component in the λ_{probe} = 685 nm signal in **Supplementary Figure 16h** provides further evidence that the aggregate sub-population assigned to the τ_2 time constant has been preferentially excited and is the primary dimer population excited at 675 nm. This is evident in the main part of **Supplementary Figure 16h** as well as in the inset of **Supplementary Figure 16h**, which is plotted for direct comparison with the inset of **Supplementary Figure 16d**. Taken together, the difference in the TA spectra obtained with λ_{pump} = 675 nm versus λ_{pump} = 620 nm, as well as the increased contribution of the τ_2 time constant to the kinetics observed at λ_{probe} = 685 nm, help confirm the heterogeneous nature of the SQ dimer solution, with both dimer aggregate structures displaying excitonic lifetimes of < 75 ps.

To test how adding a rotaxane ring to each of the dyes composing the dimer impacts excited-state quenching, we next measured the TA of a SR dimer solution, shown in **Supplementary Figure 17**. As detailed in **Supplementary Note 16**, we performed a similar investigation of pump wavelength dependence on the SR dimer solution, but, in contrast to the SQ dimer solution, the excited-state dynamics of the SR dimer are largely independent of the excitation wavelength, indicating that the population of aggregate structures in the solution is largely homogeneous, i.e., only one dimer structure contributes to the TA signal. As a result, **Supplementary Figure 17** only displays the measurement for λ_{pump} = 690 nm, which corresponds to the most intense and lowest-energy band in the steady-state absorption spectrum of the SR dimer solution. The surface plot in **Supplementary Figure 17a** and transient spectra shown in panel **b** are much simpler compared with the SQ aggregate solution (**Supplementary Figure 16**). The TA spectra of the SR dimer solution show two positive-going features between ~600-720 nm. Based on their good correspondence with the steady-state absorption spectrum, we assign these positive-going features to GSB bands of the SR dimers. As in the SQ dimer measurements, the GSB features eventually decay into a long-lived (τ > 1.4 ns) signal that closely matches the TA of a sample of SR monomer (**Supplementary Figure 15**), which, as noted above, has a ~2.9 ns lifetime, similar to that of the SQ monomer (**Supplementary Note 11**). There are also three negative-going features that we assign as ESA bands. First, an ESA band is observed near 470 nm, along with another ESA band that extends beyond the spectral range probed to >740 nm. Since these features appear in both the SR monomer and dimer solutions, we assign them to both the SR monomer and dimer populations (**Supplementary Note 15**). Finally, given the discrepancy between the GSB features and the steady-state absorption spectrum in the vicinity of ~660 nm, we identify a third ESA signal at around 660 nm that we attribute to the SR dimer. Additional support for this interpretation is related to the time dependence of the signal that we describe in more detail below. Even though there is substantial monomer signal, panels **a** and **b** show that the GSB features associated with the SR aggregate persist much longer than that of the SQ dimer, i.e., the GSB features decay into those associated with the monomer at time delays of several hundred ps, rather than within about 100 ps, as observed in the SQ aggregate.

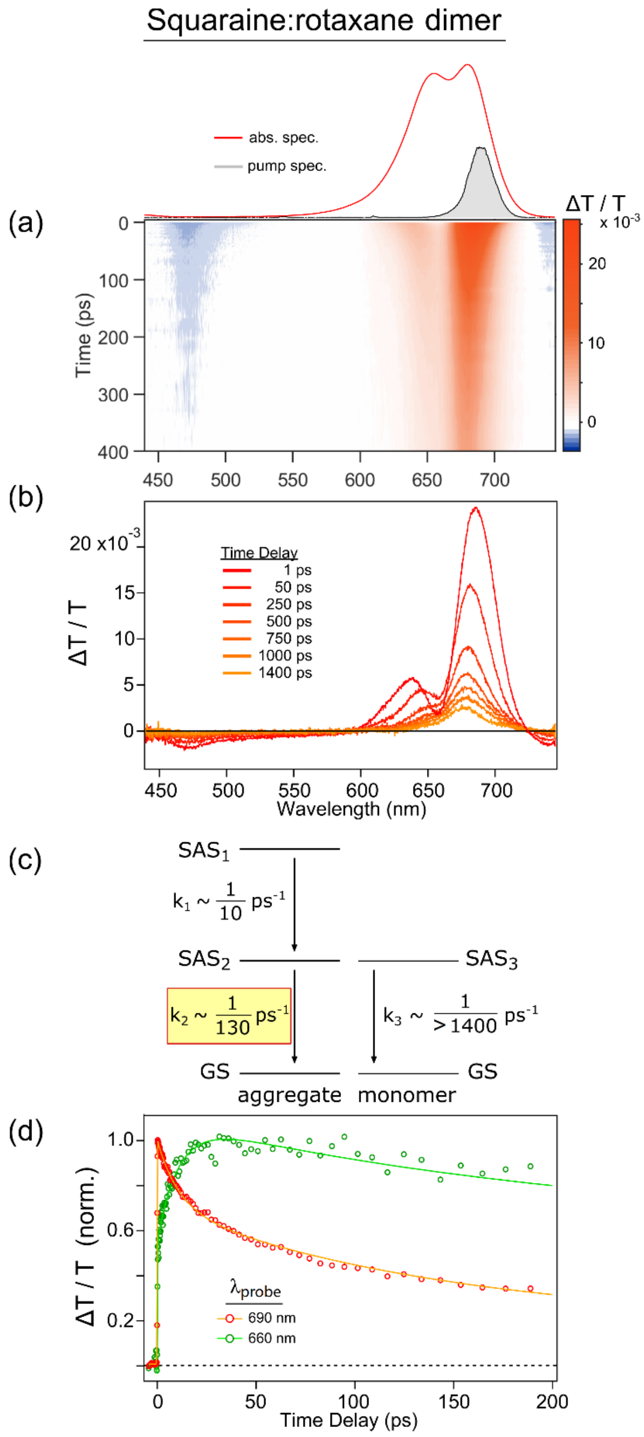

Supplementary Figure 17. Transient absorption (TA) of squaraine:rotaxane (SR) dimers templated in the form of a transverse dimer using a DNA Holliday junction. (**a**) TA surface plot. Experiment was performed with an incident pump wavelength of 690 nm and pump fluence of 19 μJ cm^{-2}. Scale bar is indicated beside the surface plot, with the corresponding steady-state absorption and pump excitation spectra included above the surface plot. (**b**) Selected representative TA spectra. Spectral time delays are indicated in the legend. (**c**) Kinetic scheme used to model the

TA dynamics via global target analysis. Rate constants (expressed as inverse time constants) associated with conversion between different components exhibiting distinct species-associated spectra (SAS_n) are shown, with the rate constant associated with the excited-state lifetime of the SR dimer structure highlighted in yellow. (**d**) TA kinetics at selected probe wavelengths (i.e., selected spectral slices of the surface plot shown in panel **a**). Each kinetics trace has been normalized to its maximum amplitude. Fits from the global target analysis (per the kinetics scheme shown in panel **c**) are overlaid on the kinetics traces.

To gain further insight into the excitonic lifetime of the SR dimer, we also performed a global target analysis on the TA of the SR dimer solution. The analysis used the three-component kinetic model shown in **Supplementary Figure 17c**. Details of and justification for the model are provided in **Supplementary Note 15**. The rate constants from the global target analysis are, again, expressed as inverse time constants (i.e., $k_n = 1/\tau_n$) and $\tau_{1\text{-}3}$ values of 10, 130, and > 1400 ps are derived. As in the analysis of the SQ aggregate measurements, the last time constant is assigned to a small sub-population of monomers. Given that the solution is largely homogeneous, as justified above, we assign the remaining two time constants to the SR dimer. The more rapid dynamics associated with the faster τ_1 ~ 10 ps time constant are evident in the signal at 660 nm in the TA spectra shown in **Supplementary Figure 17b**, which increases after a time delay of 1 ps. The slower τ_2 ~ 130 ps time constant is evident in the subsequent longer-timescale decay of the entire signal associated with the dimer, which decays into the TA spectrum of the monomer. In addition to these time-dependent changes in the spectra, the normalized kinetic traces shown in **Supplementary Figure 17d** further highlight the sequential kinetics of the SR dimer. Specifically, the faster τ_1 ~ 10 ps time constant associated with the growth of the signal at 660 nm is evident in the kinetics trace for λ_{probe} = 660 nm, whereas the slower τ_2 ~ 130 ps time constant associated with the decay of the entire SR dimer spectrum is better represented in the kinetics trace for λ_{probe} = 690 nm. Thus, we find that the dimer population in the SR solution undergoes sequential decay kinetics with a relatively fast time constant of ~10 ps followed by a longer decay of ~130 ps. Based on the complete recovery of the GSB features, we can straightforwardly assign the second time constant to the excitonic lifetime of the SR dimer. While the physical origin of the first time constant observed in both SQ and SR dimers is currently unclear, as is discussed in **Supplementary Note 15**, the assignment of the second time constant is unambiguously associated with the excitonic lifetime. The data clearly indicate that the excitonic lifetime of the SR dimer is significantly extended as compared with the SQ dimers. Specifically, compared with the ~20 and ~60-80 ps

excitonic lifetimes measured for the two dimer structures present in the SQ dimer solution, an extended lifetime of ~130 ps lifetime is measured for the single dimer structure found in the SR dimer solution.

<u>Supplementary Note 15</u>: Four- and three-component kinetic schemes best model the squaraine and squaraine:rotaxane dimer solutions, respectively

The transient absorption of the DNA-templated squaraine (SQ) and squaraine:rotaxane (SR) dimer solutions presented in **Fig. 5** in the main text and in **Supplementary Figures 16** and **17**, respectively, were analyzed via a global target analysis based on the kinetic models presented in **Supplementary Figure 18**.

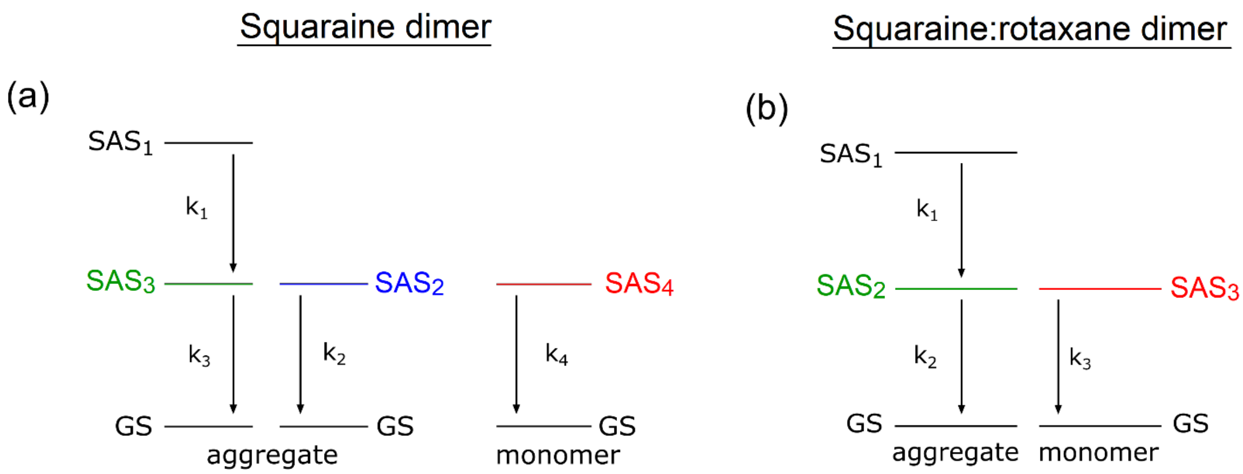

Supplementary Figure 18. Kinetic models used in the global target analysis of the transient absorption (TA) of commercially available squaraine (SQ) and squaraine:rotaxane (SR) dyes Square 660 and SeTau 670, respectively, templated via a four-armed Holliday junction (HJ) in the form of a transverse dimer. (**a**) Four-component model showing the sequential decay of SQ dimer population (SAS_1, SAS_3), a shorter-lived sub-population of SQ dimers (SAS_2) decaying in parallel, as well as long-lived monomer population (SAS_4). (**b**) Three-component kinetic model showing the sequential decay of SR dimers (SAS_1, SAS_2) with an additional monomer population (SAS_3) that decays in parallel.

The following parts of this section discuss the main justifications for the kinetic models employed for each dimer solution. The data is initially modeled using a basis of two components, and the remaining number of components in each scheme are justified mathematically by examining the goodness of the fit to the data. We make a physical assignment for each component, and we present and discuss evidence supporting the physical assignments. We conclude this supporting section with a summary of the results of the global target analysis.

The simplest kinetic models that best describe the data contain four decay components in the TA of the SQ dimer solution and three components for the SR dimer solution. **Supplementary**

Figure 19 displays selected kinetic traces from the TA of the SQ dimer solution with pump wavelengths of 620 and 675 nm and the SR dimer solution with a pump wavelength of 690 nm, along with the fits from a global target analysis with a varying number of components.

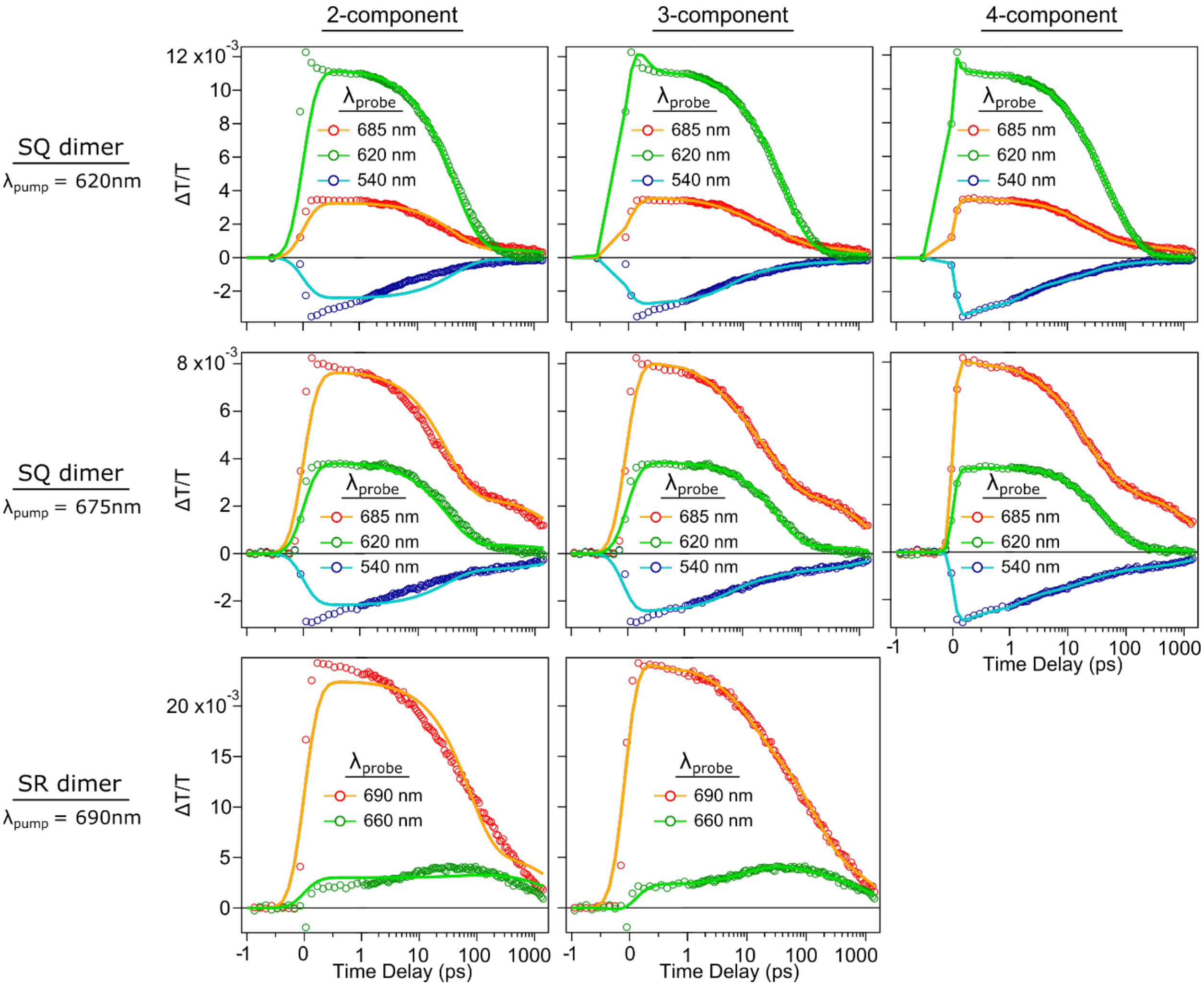

Supplementary Figure 19. Selected TA kinetic traces and corresponding fits according to a global target analysis for commercially available squaraine (SQ) and squaraine:rotaxane (SR) dyes templated via a four-armed Holliday junction (HJ) in the form of a transverse dimer. Data are plotted as circles and fits overlay the data as lines for 2-component (left column), 3-component (middle column), and 4-component (right column) global target analysis. Results are presented for SQ dimer solution at 620 nm (top row) and 675 nm (middle row) excitation wavelengths, and the SR dimer solution at 690 nm (bottom row). For these measurements, the dimer solutions were not subjected to polyacrylimide gel electrophoresis due to the high concentrations and volumes required for the TA measurements.

We use two components for the initial model, with one component accounting for a dimer population and a second component accounting for a small monomer sub-population. We include

a small monomer sub-population because fluorescence excitation measurements indicate that an appreciable sub-population of monomers exists in both SQ and SR dimer solutions (see **Supplementary Note 12**). We further confirmed the presence of a sub-population of monomers in the TA measurements, where at long time delays the TA spectrum in the dimer solutions is very similar to the TA spectrum of the monomers (**Supplementary Figure 15**). **Supplementary Figure 19** shows that the fits from the global analysis assuming a two-component scheme are inadequate to describe the data. For the SQ dimer solution TA measurements, this is most evident in deviations between the data and the fits for the probe wavelengths of 540 and 685 nm; for the SR dimer solution TA measurements, the deviations are most noticeable for probe wavelengths of 660 and 690 nm. In the case of the TA measurements of the SR dimer solution, the addition of a third component in the kinetic scheme enables the fit to much better model the data. Thus, three components are mathematically justified for the TA measurements of the SR dimer solution pumped at 690 nm. For the SQ dimer solution, on the other hand, there are significant deviations between the fits and the data at both pump wavelengths, particularly for the signals observed at probe wavelengths of 540 and 685 nm. Thus, a fourth component was included in the model, which **Supplementary Figure 19** shows enables the fits to model the data more accurately. In conclusion, starting from a basis of two components accounting for dimer and monomer sub-populations, four components were found to be mathematically justified to model the TA measurements of the SQ dimer solutions at pump wavelengths of 620 and 675 nm, while three components were found to be mathematically justified to model the TA measurements of the SR dimer solutions at a pump wavelength of 690 nm.

We proceed to discuss the physical assignment of the different components in the kinetic schemes for the SQ and SR dimer solutions. As described above, we assign the longest-lived component to a sub-population of monomers present in both SQ and SR dimer solutions. Further evidence in support of this interpretation is shown in **Supplementary Figure 20**, which displays the fourth and third species-associated spectra (SAS) obtained from the TA of the SQ and SR solutions, respectively, compared with the SAS of the corresponding monomer solution fit using a single-component analysis.

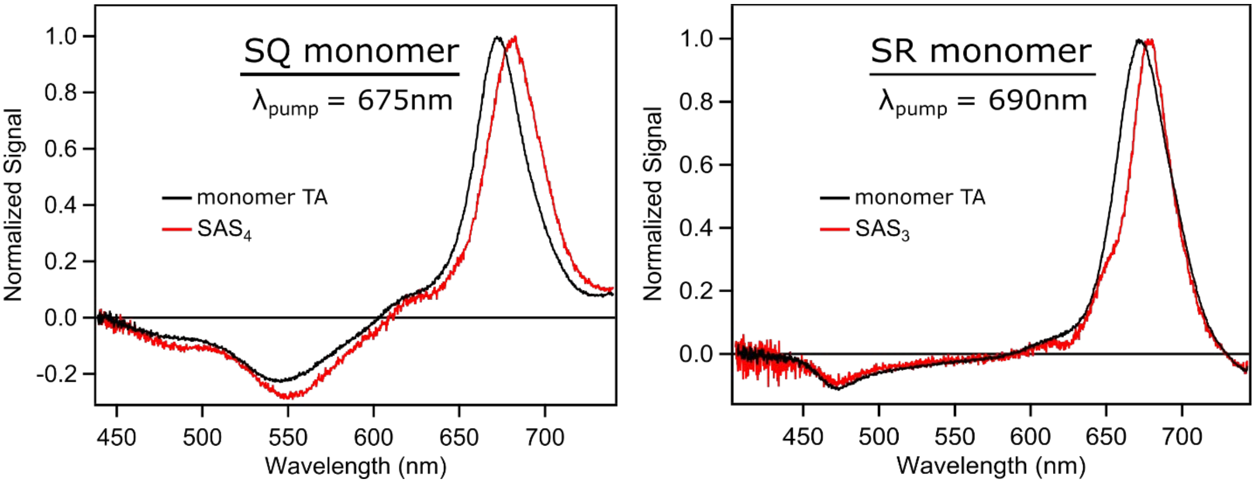

Supplementary Figure 20. Comparison of species-associated spectra (SAS) obtained from measurements of DNA-templated SQ and SR monomer and dimer solutions. The longest-lived decay component obtained from a four- and three-component analysis of the TA of the SQ and SR dimer solutions, respectively, is shown in red. The result of a single-component analysis of the TA of the SQ and SR monomer solutions is shown in black. The results associated with SQ and SR solutions are plotted on the left and right, respectively.

The reasonable agreement between the longest-lived SAS of the SQ and SR dimer solutions and the SAS of the corresponding monomer solutions confirms the presence of a monomer sub-population in both dimer solutions. Additionally, we note that the time constants calculated from the single-component analysis of the TA of the monomer of $\tau \sim 1700$ ps (SQ) and $\tau \sim 1400$ ps (SR) differ from the monomer lifetimes of ~2.9 ns measured via TCSPC (**Supplementary Note 11**). This result is not entirely unexpected, since the longest time delay possible in the TA experiment is 1.4 ns (due to limitations of the length of the translation stage). To account for these discrepancies in the global target analysis of the dimer solutions, we fix the time constants associated with the longest-lived decay components in the dimer solutions to the time constants determined from a global target analysis of the monomer solutions performed according to the same experimental conditions. That is, we fix $\tau_4 \sim 1700$ ps in the SQ dimer solution and $\tau_3 \sim 1400$ ps in the SR dimer solution.

We next proceed to discuss the physical assignment of the remaining two components in the kinetic scheme for the SR dimer solution. Having assigned one of the three components in the model used for the SR dimer solution as a monomer sub-population, the remaining two components could arise from: (i) the sequential decay of a single population of dimer structures, which assumes the solution is homogeneous, or (ii) the parallel decay of two populations of dimer structures, which assumes the solution is heterogeneous. Because the SR dimer solution is found

to be largely homogeneous (**Supplementary Note 16**), we conclude that the remaining components correspond to the sequential decay of a single population of SR dimer structures. The resultant SAS are displayed in **Supplementary Figure 21**.

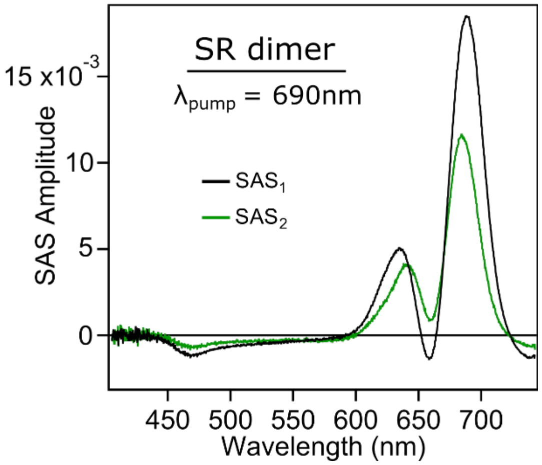

Supplementary Figure 21. Species-associated spectra (SAS) resulting from a global target analysis of the TA of a commercially available squaraine:rotaxane (SR) dye templated via a four-armed Holliday junction (HJ) in the form of a transverse dimer. A three-component kinetic scheme involving a sequential decay of the dimers was used, where SAS_1 converts into SAS_2 with a time constant of τ_1 ~ 10 ps, and SAS_2 decays with a lifetime of τ_2 ~ 130 ps.

Two components, i.e., SAS_1 and SAS_2, are observed with associated lifetimes τ_1 ~ 10 ps and τ_2 ~ 130 ps. As described in the main text, the complete recovery of the ground-state bleach features associated with SAS_2 in the TA measurement enables a straightforward assignment of the τ_2 component as the excitonic lifetime of the SR dimer structure. As a result, the rapid decay of SAS_1 into SAS_2 may be associated with some form of excimer relaxation [18–20] or possibly biexcitonic decay [21–23], but may also include vibrational cooling or solvent relaxation.

We next proceed to discuss the physical assignment of the remaining three (of the four total) components in the kinetic scheme for the SQ dimer solution. Based on the drastically different TA results obtained at different pump wavelengths that indicate the SQ dimer solution is heterogeneous (**Supplementary Figure 16**), we attribute at least two of the three components to a parallel decay mechanism. An evaluation of the decay-associated spectra (DAS) and SAS resulting from the global target analysis is useful in identifying the spectral features of the heterogeneous populations in the SQ dimer solution, which helps in the assignment of the components in the parallel decay mechanism. The analysis extracts the DAS as spectral traces of the TA that decay with the fitted time constants, and then obtains the physically relevant SAS as linear combinations

of the DAS according to the sequential or parallel mechanism(s) of the kinetic model [16,17]. For example, for a model in which all components correspond to separate populations that decay independently of each other (i.e. in parallel), the calculated DAS would be identical to the SAS of each population. The DAS obtained from the analysis of the SQ dimer solution at λ_{pump} = 620 nm and λ_{pump} = 675 nm are shown in **Supplementary Figures 22a** and **22d**, respectively. Comparing the DAS with the TA from **Supplementary Figures 16b** and **16f**, it is clear that the positive-going features of the primary decay component observed in the TA at λ_{pump} = 675 nm have the best agreement with DAS_2, particularly in **Supplementary Figure 22d**, while the TA from the primary component at λ_{pump} = 620 nm most closely resembles DAS_3. As a result, we assign DAS_2 and DAS_3 as the two major components of the parallel decay mechanism. Therefore, SAS_2 and SAS_3 in the lower panels of **Supplementary Figure 22** are identical to the corresponding DAS, and are identified as two separate populations of SQ dimer which undergo excitonic decay lifetimes of τ_2 ~ 15-18 ps and τ_3 ~ 58-75 ps, respectively.

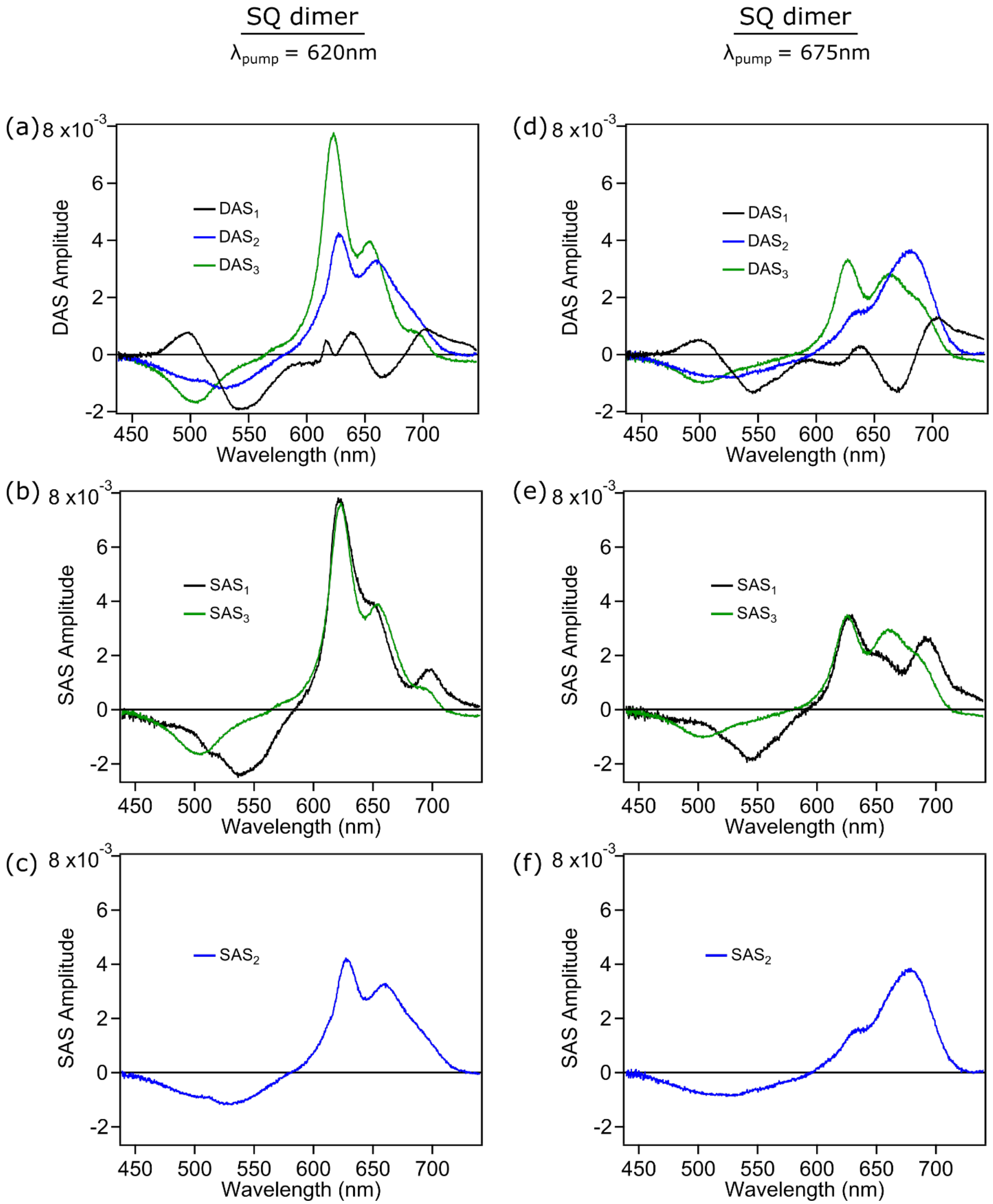

Supplementary Figure 22. Results from global target analysis of the TA at multiple excitation wavelengths of a commercially available squaraine (SQ) dye templated via a four-armed Holliday junction (HJ) in the form of a transverse dimer. (**a** and **d**) Decay-associated spectra (DAS) corresponding to aggregate time constants of $\tau_1 \sim 1$ ps (black), $\tau_2 \sim$ 15-18 ps (blue), and $\tau_3 \sim$ 58-75 ps (green). (**b** and **e**) Species-associated spectra (SAS) corresponding to the sequential decay

mechanism that is associated with excitation of the shorter-wavelength population, where SAS_1 converts into SAS_3 with a time constant of τ_1 ~ 1 ps, and SAS_2 decays with a lifetime of τ_3 ~ 58-75 ps. (**c** and **f**) SAS corresponding to the parallel decay associated with excitation of the longer-wavelength population, where SAS_2 decays with a lifetime of τ_2 ~ 15-18 ps.

Having identified that two of the components are associated with dimer structural heterogeneity in the SQ dimer solution, we next focus on the physical assignment of the remaining component. As noted above, the other components have been assigned to time constants $\tau_{2\text{-}4}$; therefore, the first component (DAS_1) associated with τ_1 ~ 1 ps either corresponds to the parallel decay of a third dimer sub-population or a sequential decay mechanism associated with one of the dimer populations. Given that the spectrum of DAS_1 is composed of multiple positive- and negative-going signals with no clear analogue in the steady-state absorption and TA spectra of the SQ dimer, it is fairly unlikely that DAS_1 is associated with a separate population decaying in parallel. Thus, we assign the remaining component associated with τ_1 to be part of a sequential decay mechanism, as in the SR dimer. We attribute the sequential decay to the first component decaying into the third component, based on the fact that, between time delays of 1 and 5 ps in **Supplementary Figures 16b** and **16f**, it is clear that the increase of the negative-going amplitude at 540 nm, which is apparent in DAS_1 and associated with the decay of the first component, is more intense at λ_{pump} = 620 nm than at λ_{pump} = 675 nm. Because the dimer structure that absorbs at 620 nm exhibits more TA amplitude in the spectral regions associated with DAS_1 and DAS_3, we assign the first and third components as a sequential decay mechanism of the SQ dimer population associated with SAS_3. Similar to the sequential process in the SR dimer solution, the similarities between the spectra in **Supplementary Figure 22b** suggest that the decay of the first component into the third component in the SQ dimer solution may correspond to a process involving solvent relaxation, vibrational cooling, excimer relaxation, or biexcitonic decay.

In conclusion, the four- and three-component kinetic schemes provide effective models to describe the TA of the SQ and SR dimer solutions, respectively. We used an initial two-component kinetic scheme consisting of dimer and monomer sub-populations to model the data, and mathematically found that four- and three-component kinetic schemes were needed to appropriately model the TA of the SQ and SR dimer solutions, respectively. Because we found the SR dimer solution to be largely homogeneous, we assigned two of the three components in the kinetic scheme for the TA of the SR dimer solution to a single dimer population. We calculated time constants of ~10 ps and ~130 ps, with the latter being associated with the excitonic lifetime

of the SR dimer population. The more complicated photophysics of the SQ dimer solutions was handled in two parts. First, we assigned two of the three remaining components to two populations of dimer structures decaying in parallel, which exhibit excitonic lifetimes of ~20 and ~60-80 ps. Second, we assigned the first component of the model to the sequential decay of the population of dimer structures absorbing most strongly at 620 nm.

<u>Supplementary Note 16</u>: Transient absorption of squaraine:rotaxane dimer solution is largely invariant to pump wavelength

To evaluate the squaraine:rotaxane (SR) dimer solution for potential dimer structural heterogeneity, we performed TA measurements at two different pump wavelengths. Two pump wavelengths of 652 and 690 nm were chosen to selectively excite the two strongest absorption bands in the steady-state absorption spectrum of the SR dimer solution. In principle, the two absorption bands could arise from: (i) a homogeneous solution composed of just one dimer structure with an oblique packing arrangement, which exhibits excitonically blue- and red-shifted bands (with respect to the monomer), or (ii) a heterogeneous solution composed of two dimer structures, one exhibiting an H-like packing arrangement with an overall blue-shifted absorption spectrum and another exhibiting a J-like packing arrangement with an overall red-shifted absorption spectrum.

Surface plots of the TA measurements are shown in **Supplementary Figures 23a** and **23c**, along with the steady-state absorption spectra and pump excitation profiles for reference.

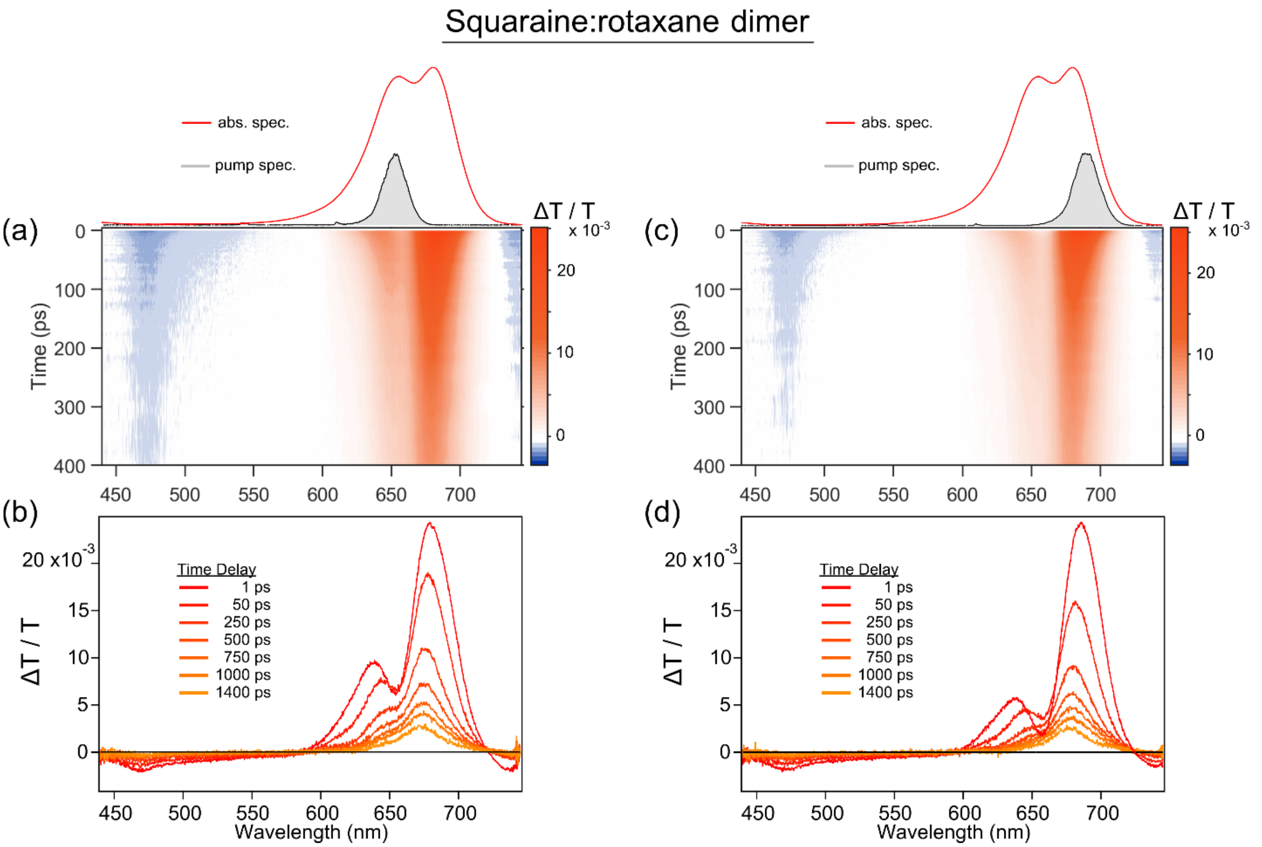

Supplementary Figure 23. Transient absorption (TA) of squaraine:rotaxane (SR) dimers templated in the form of a transverse dimer using a DNA Holliday junction. (**a** and **c**) TA surface plots. Experiments were performed with incident central pump wavelengths of 652 and 690 nm and pump fluences of 16 and 19 μJ cm^{-2}, respectively. Scale bar is indicated beside each plot. The corresponding steady-state absorption and pump excitation spectra are included above the surface plots. (**b** and **d**) Selected TA spectra. Spectral time delays are indicated in the legend.

Overall, the two surface plots, as well as the selected spectral cuts at various time delays displayed in panels **b** and **d**, are qualitatively similar. Positive- and negative-going TA features are observed at identical probe wavelengths and decay on roughly the same timescales.

The only visible discrepancy is observed in the region of ~660 nm, where positive- and negative-going features overlap. As described in the main text, we attribute the positive- and negative-going signals to ground-state bleach (GSB) and excited-state absorption (ESA) features, respectively, of the dimer structure. In the case of 690 nm excitation, the initial amplitude of the ESA feature, evident in the TA spectrum at 1 ps time delay, is much larger. At longer time delays (i.e., by 50 ps), the ESA feature has decayed appreciably, such that the positive-going signals associated with the GSB of the dimer structure more closely resemble the steady-state absorption spectrum. In the case of 652 nm excitation, the overlapping ESA band at 660 nm is noticeably less intense in the TA spectrum at 1 ps time delay. Because of the lower initial amplitude, the subsequent decay is less obvious. However, at a time delay of 50 ps, the TA spectra look very similar for the measurements at the two different central pump wavelengths.

There are several possible ways to explain the slight discrepancies observed in the TA of the SR dimer solutions at the two different pump wavelengths. The possibilities include: (i) the SR dimer solution exhibits a small extent of dimer structural heterogeneity, wherein the additional dimer structure has a GSB feature in the vicinity of 660 nm, (ii) sub-picosecond dynamics associated with excess pump energy are causing complicated structural relaxation dynamics that cause the 1-to-10 picosecond timescale TA spectra to look different, or (iii) a systematic error owing to non-negligible dynamic pump scatter in the vicinity of the pump wavelength. Given that we find nearly identical decays in both experiments, calculating time constants of $\tau_1 \sim 10$ ps and $\tau_2 \sim 130$ ps at 690 nm excitation, and $\tau_1 \sim 10$ ps and $\tau_2 \sim 140$ ps for the 652 nm pump wavelength (see the global target analysis discussed in detail in **Supplementary Note 15**), dimer structural heterogeneity is unlikely to be a primary contributor to the discrepancy. This then leaves possibility (ii) or (iii) to explain the discrepancy, neither of which are related to dimer structural heterogeneity. Thus, the general invariance of the TA spectra and time constants to incident pump wavelength

(as compared with the striking changes in the SQ dimer solution, see **Supplementary Figure 16**) suggests that the SR dimer solution is largely homogeneous, i.e., is composed largely of a single population of SR dimer structures.

Supplementary Note 17: Rotaxane ring remains attached to squaraine dye after attachment to DNA and of the nanostructure in aqueous solution

To determine if the squaraine:rotaxane (SR) complex was still present after the dyes are attached to DNA via the amidation reaction (see **Supplementary Note 1**), we present the matrix assisted laser desorption ionization-time of flight (MALDI-TOF) mass spectrum provided by Bio-Synthesis for quality control. The MALDI-TOF mass spectrum of the SeTau-670 dye attached to the custom DNA "C" strand (**Supplementary Note 3**) is shown in **Supplementary Figure 24**. Based on the molecular weights (MWs) of the expected components, we expect to see the DNA-SeTau-670 dye construct in the vicinity of the red arrow in the Figure. Although the exact chemical structure of the SeTau-670-NHS dye is proprietary, we can estimate that, based on the MW of SeTau-670-NHS reported on SETA's website (1880.09 g/mol) [1], the MW of the unlabeled custom oligonucleotide "C" strand (7757 g/mol), the molecular structure of the serinol linker (MW of ca. 222 g/mol; **Supplementary Note 1**), that of the N-hydroxysuccinimide substituent (ca. 114 g/mol), and the minimum possible MW of the rotaxane ring (ca. 540 g/mol, assuming n=0, X=O, R=H; i.e. chemical formula $C_{28}H_{24}N_6O_6$), the DNA-SeTau-670 construct has m/z ~ 9745 (red arrow) and a DNA-squaraine precursor construct would have m/z ≤ 9205 (blue arrow). Not only does **Supplementary Figure 24** show that there are ions that were measured in the vicinity of 9745 g/mol, but also the base peak of the mass spectrum has m/z = 9359, which indicates that the most common fragment ions observed in the MALDI-TOF mass spectrum are noticeably more massive than the fragment ions that would result if the squaraine rotaxane had undergone decomplexation. Therefore, the significant presence of fragment ions more massive than the DNA-squaraine precursor construct provides strong evidence that the SR complex is still intact.

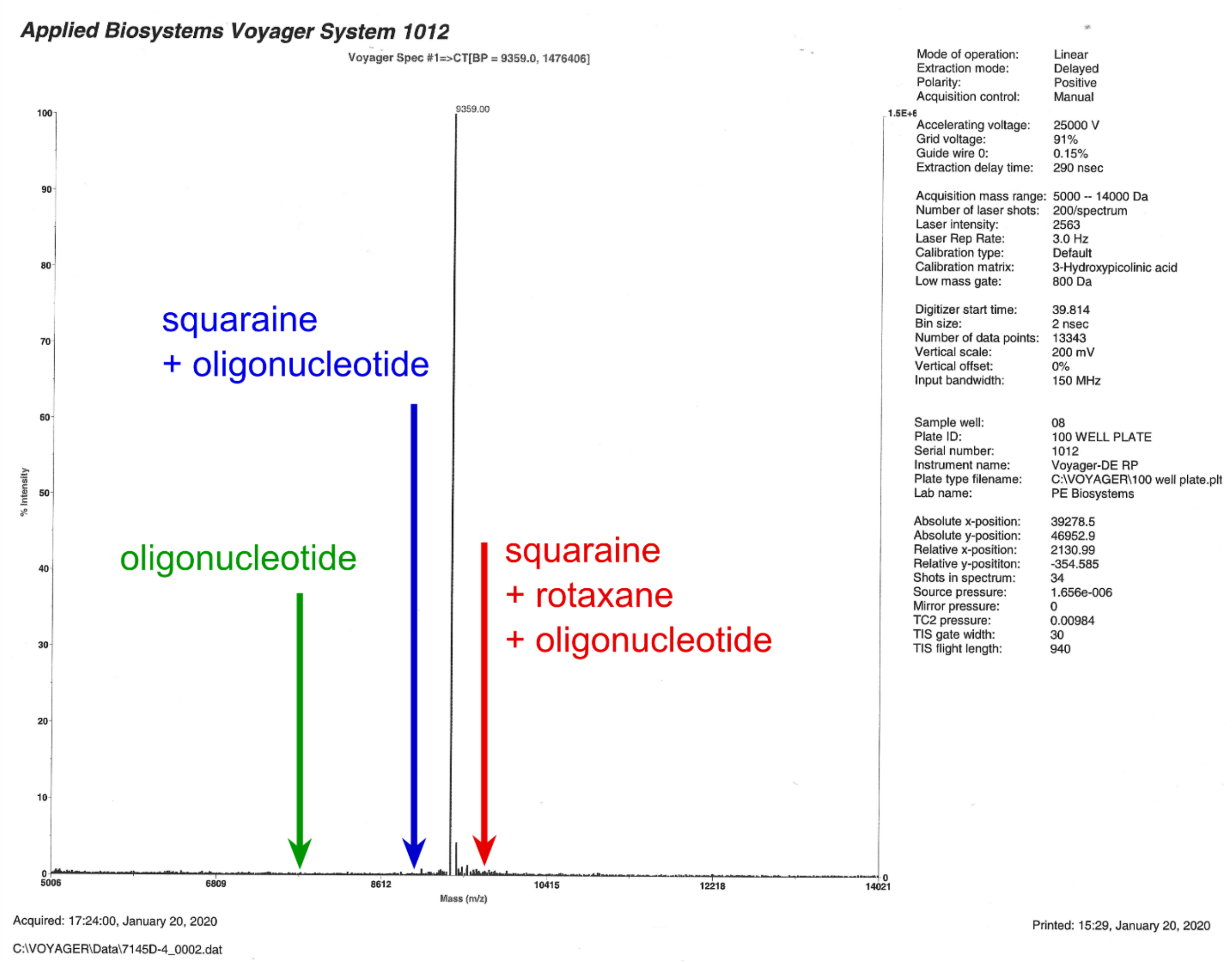

Supplementary Figure 24. MALDI-TOF mass spectrum of the SR dye attached to "C" strand DNA (see **Supplementary Note 3** for full sequence). Based on molecular weights, the presence of the SR dye attached to DNA should be observed near the red arrow. The blue arrow indicates the molecular weight of the squaraine precursor attached to DNA, based on a lower-bound estimate of the molecular weight of the rotaxane. The green arrow corresponds to the molecular weight of the DNA strand only. DNA attachment and mass spectrometry were carried out by Bio-Synthesis.

Having confirmed that the SR complex is still present after attachment of the SR dye to DNA, we now demonstrate that the optical properties of the SR complex are unchanged after the DNA-dye construct is solvated in aqueous solution and hybridized to form a DNA HJ. To this end, we have measured the absorption spectra of the precursor squaraine dye and SR complex in aqueous solution, shown in **Supplementary Figure 25**, and compared it with that of the SR monomer sample. The squaraine precursor exhibits an extensive blue-shift of 28 nm (645 cm^{-1}). As such, if the complex were unstable when the DNA-dye construct is hydrated in water, we could expect the emergence of a feature at 645 nm. Upon comparing the absorption spectrum of the

DNA-dye construct hydrated in aqueous solution with the SR complex dissolved in aqueous solution, we find the results match up nearly identically, and we do not see a prominent feature in the vicinity of 645 nm. Therefore, we conclude that the SR complex remains intact even following the solvation of the DNA-dye construct in aqueous solution.

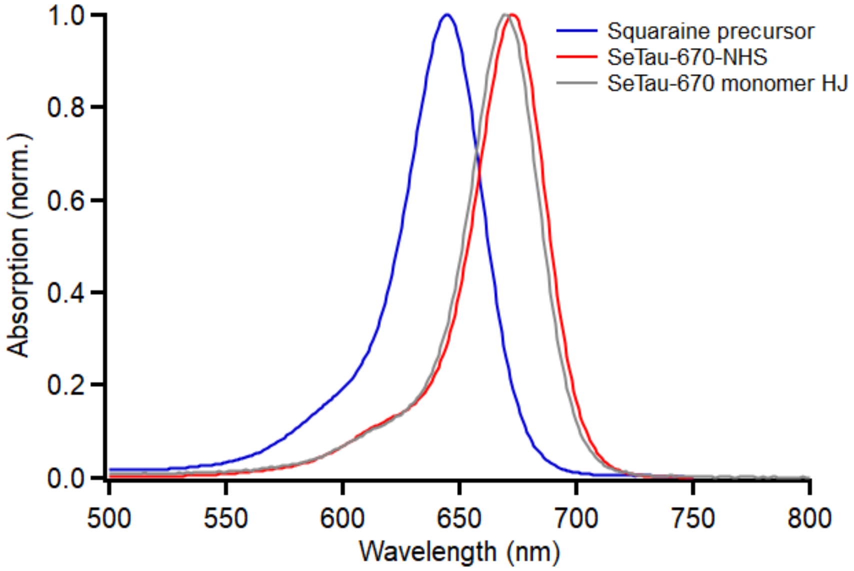

Supplementary Figure 25. Steady-state absorption spectra of aqueous solutions of the squaraine precursor of the SR dye (blue), the SeTau-670-NHS dye itself (red), and the SR dye templated using a DNA HJ in the form of a monomer (grey). The SeTau-670-NHS and squaraine precursor dyes are each in solutions of 67mM phosphate buffer (pH 7.4), and the SR monomer is in a buffer solution of 1×TBE + 15 mM $MgCl_2$.

Supplementary Note 18: Characterization of the temporal pulsewidth of the pump beam in the transient absorption measurement

We measured the duration of the pump pulse (650 nm) at the sample position by performing an autocorrelation measurement using polarization gating [15]. As described in our previous report [13], the pump and probe beams were set to relative polarizations of 45° and focused into a 2 mm pathlength quartz cuvette. The pulses were tuned to overlap at the sample in time and in space, and the relative time delay between the pump and the probe pulses was varied with a mechanical linear translation stage. A Gaussian fit of the autocorrelation signal determined the FWHM to be ca. 180 fs, as shown in **Supplementary Figure 26**.

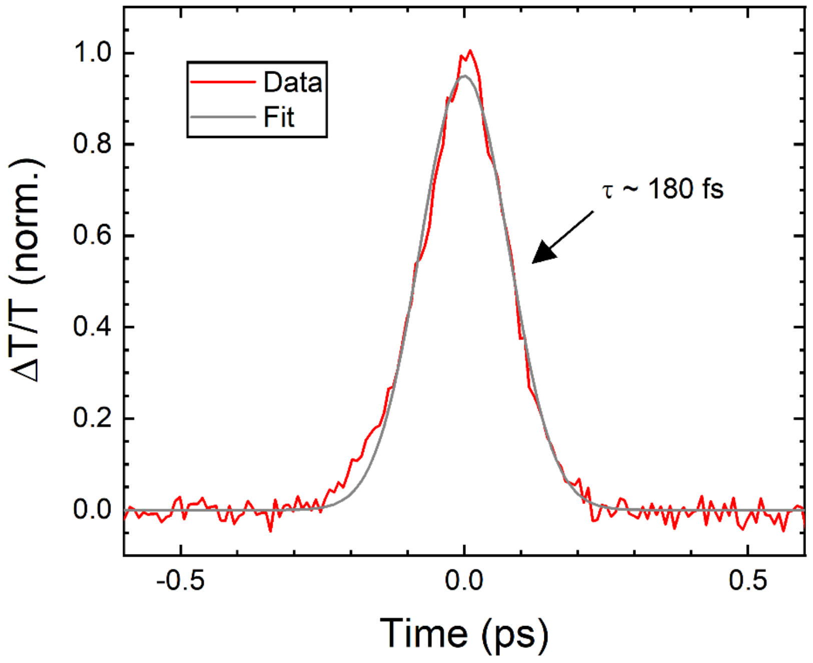

Supplementary Figure 26. Autocorrelation measurement of the pulse duration of the pump beam. The temporal intensity (black) is fit to a Gaussian (red), and the FWHM of 180 fs is indicated.

<u>Supplementary Note 19</u>: Spectrum of the white-light continuum used as the probe beam in the transient absorption measurement

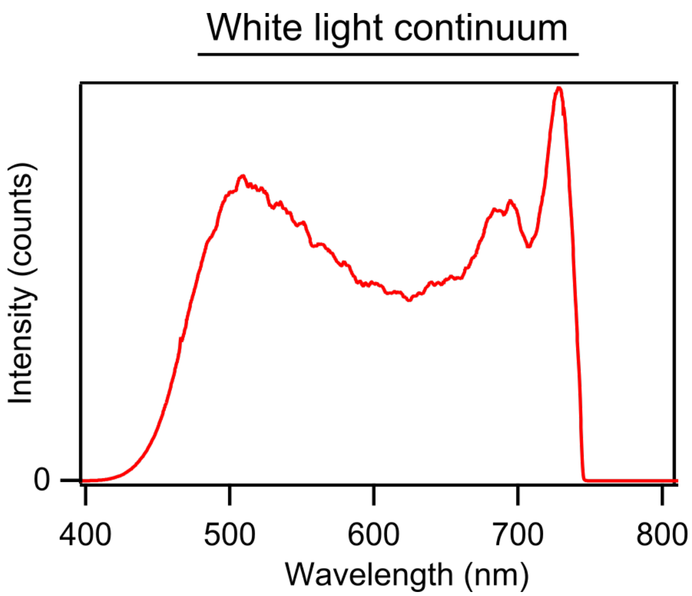

Supplementary Figure 27. Representative spectrum of the white light continuum used as the probe beam in the transient absorption measurements.

Supplementary references

1. K9-4169 SeTau-670-NHS. https://www.setabiomedicals.com/k9-4169.html.
2. Shida, T., Iwasaki, H., Shinagawa, H. & Kyogoku, Y. Characterization and Comparison of Synthetic Immobile and Mobile Holliday Junctions. *J Biochem* **119**, 653–658 (1996).
3. Carr, C. E. & Marky, L. A. Melting Behavior of a DNA Four-Way Junction Using Spectroscopic and Calorimetric Techniques. *J. Am. Chem. Soc.* **139**, 14443–14455 (2017).
4. Mass, O. A. *et al.* Exciton Delocalization in Indolenine Squaraine Aggregates within DNA Holliday Junction Scaffolds. *J. Phys. Chem. B* **124**, 9636–9647 (2020).
5. Cannon, B. L. *et al.* Coherent Exciton Delocalization in a Two-State DNA-Templated Dye Aggregate System. *J. Phys. Chem. A* **121**, 6905–6916 (2017).
6. Cannon, B. L. *et al.* Large Davydov Splitting and Strong Fluorescence Suppression: An Investigation of Exciton Delocalization in DNA-Templated Holliday Junction Dye Aggregates. *J. Phys. Chem. A* **122**, 2086–2095 (2018).
7. Fothergill, J. W., Hernandez, A. C., Knowlton, W. B., Yurke, B. & Li, L. Ab Initio Studies of Exciton Interactions of Cy5 Dyes. *J. Phys. Chem. A* **122**, 8989–8997 (2018).
8. Kühn, O., Renger, T. & May, V. Theory of exciton-vibrational dynamics in molecular dimers. *Chemical Physics* **204**, 99–114 (1996).
9. Mukamel, S. *Principles of Nonlinear Optical Spectroscopy*. (Oxford University Press, 1995).
10. Hamm, P. Principles of Nonlinear Optical Spectroscopy: A Practical Approach. 77.
11. Berg, J. M. *et al. Biochemistry*. (W H Freeman, 2002).
12. Cunningham, P. D. *et al.* Optical Properties of Vibronically Coupled Cy3 Dimers on DNA Scaffolds. *J. Phys. Chem. B* **122**, 5020–5029 (2018).
13. Huff, J. S. *et al.* DNA-Templated Aggregates of Strongly Coupled Cyanine Dyes: Nonradiative Decay Governs Exciton Lifetimes. *J. Phys. Chem. Lett.* **10**, 2386–2392 (2019).
14. von Hippel, P. H., Johnson, N. P. & Marcus, A. H. Fifty years of DNA ‘breathing’: Reflections on old and new approaches. *Biopolymers* **99**, 923–954 (2013).
15. Trebino, R. *et al.* Measuring ultrashort laser pulses in the time-frequency domain using frequency-resolved optical gating. *Review of Scientific Instruments* **68**, 3277–3295 (1997).
16. Holzwarth, A. R. Data Analysis of Time-Resolved Measurements. in *Biophysical Techniques in Photosynthesis* (eds. Amesz, J. & Hoff, A. J.) 75–92 (Springer Netherlands, 1996). doi:10.1007/0-306-47960-5_5.
17. van Stokkum, I. H. M., Larsen, D. S. & van Grondelle, R. Global and target analysis of time-resolved spectra. *Biochimica et Biophysica Acta (BBA) - Bioenergetics* **1657**, 82–104 (2004).
18. Sung, J., Kim, P., Fimmel, B., Würthner, F. & Kim, D. Direct observation of ultrafast coherent exciton dynamics in helical π-stacks of self-assembled perylene bisimides. *Nat Commun* **6**, 8646 (2015).
19. Mauck, C. M., Young, R. M. & Wasielewski, M. R. Characterization of Excimer Relaxation via Femtosecond Shortwave- and Mid-Infrared Spectroscopy. *J. Phys. Chem. A* **121**, 784–792 (2017).
20. Pensack, R. D., Ashmore, R. J., Paoletta, A. L. & Scholes, G. D. The Nature of Excimer Formation in Crystalline Pyrene Nanoparticles. *J. Phys. Chem. C* **122**, 21004–21017 (2018).
21. Spano, F. C. & Mukamel, S. Cooperative nonlinear optical response of molecular aggregates: Crossover to bulk behavior. *Phys. Rev. Lett.* **66**, 1197–1200 (1991).
22. D’Avino, G., Terenziani, F. & Painelli, A. Aggregates of Quadrupolar Dyes: Giant Two-Photon Absorption from Biexciton States. *J. Phys. Chem. B* **110**, 25590–25592 (2006).

23. Zheng, C., Zhong, C., Collison, C. J. & Spano, F. C. Non-Kasha Behavior in Quadrupolar Dye Aggregates: The Red-Shifted H-Aggregate. *J. Phys. Chem. C* **123**, 3203–3215 (2019).
